# Cluster Formation in Solutions of Polyelectrolyte Rings

**DOI:** 10.1021/acsnano.3c06083

**Published:** 2023-09-20

**Authors:** Roman Staňo, Jan Smrek, Christos N. Likos

**Affiliations:** † Faculty of Physics, University of Vienna, Boltzmanngasse 5, 1090 Vienna, Austria; ‡ Vienna Doctoral School in Physics, University of Vienna, Boltzmanngasse 5, 1090 Vienna, Austria

**Keywords:** ring polymers, polyelectrolytes, slow dynamics, DNA mini-rings, clustering, threading, molecular dynamics

## Abstract

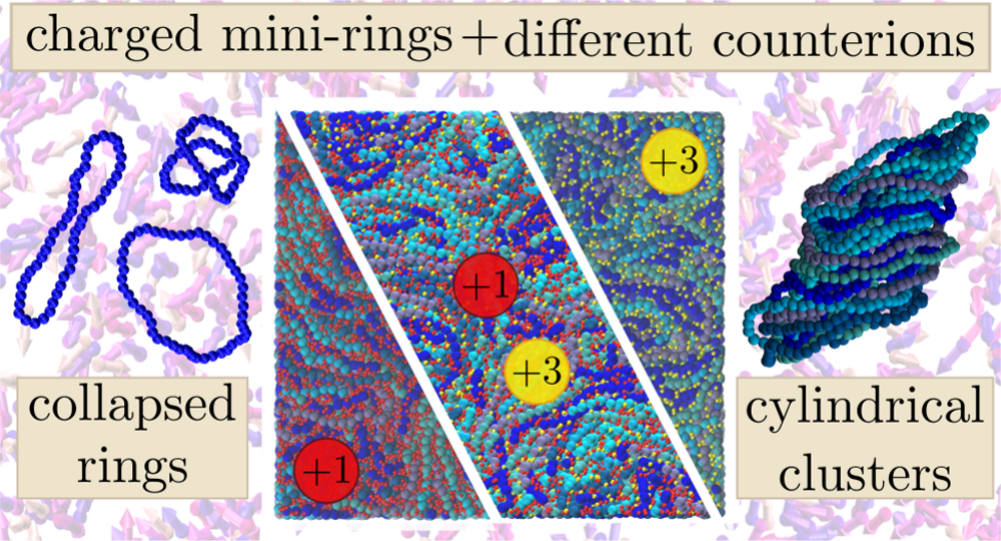

We use molecular dynamics simulations to explore concentrated solutions of semiflexible polyelectrolyte ring polymers, akin to the DNA mini-circles, with counterions of different valences. We find that the assembly of rings into nanoscopic cylindrical stacks is a generic feature of the systems, but the morphology and dynamics of such a cluster can be steered by the counterion conditions. In general, a small addition of trivalent ions can stabilize the emergence of clusters due to the counterion condensation, which mitigates the repulsion between the like-charged rings. Stoichiometric addition of trivalent ions can even lead to phase separation of the polyelectrolyte ring phase due to the ion-bridging effects promoting otherwise entropically driven clustering. On the other hand, monovalent counterions cause the formation of stacks to be re-entrant with density. The clusters are stable within a certain window of concentration, while above the window the polyelectrolytes undergo an osmotic collapse, disfavoring ordering. The cluster phase exhibits characteristic cluster glass dynamics with arrest of collective degrees of freedom but not the self-ones. On the other hand, the collapsed phase shows arrest on both the collective and single level, suggesting an incipient glass-to-glass transition, from a cluster glass of ring clusters to a simple glass of rings.

Understanding the role of topology in ring polymer solutions and melts is among the most active open problems in polymer science.^1−3^ Topological invariants stemming from noncrossability of polymer strands bring about highly specific properties, differentiating polymers with ring architecture from their linear chain counterparts. In pursuit of a theoretical description, much attention has been paid to asymptotically long rings^4^ which, for example, display distinct viscoelastic properties.^5,6^ However, rings of intermediate lengths are even more relevant in technological^7,8^ and biological applications^9−11^ for several reasons. First, the interplay of the fixed circular topology with the stiffness^12^ offers freedom in tuning the resulting effective anisotropic and soft potentials.^13,14^ This, in turn, allows for the emergence of a range of dynamic behaviors and structural properties, which can be harvested in nanomachines^15,16^ or in mechanically interlocked materials.^7,8,17−19^ Second, the circular form of DNA is strongly present in nature, exemplified by kinetoplast,^20,21^ plasmid,^22,23^ and extra-chromosomal DNAs,^24^ typically found in a range of concentration regimes of the intracellular environment and impacting the genome organization and biological function of a spectrum of organisms, including humans.^25−27^ Third, while DNA nanotechnology^28^ is based on the sequence specificity, the geometrical and topological features^29,30^ of the DNA are only recently being recognized as an additional important control parameter for mechanical and structural properties of the assembled structures and complex fluids.^31,32^ Nevertheless, in topology-oriented research, the facts that DNA is a polyelectrolyte^33^ with high charge density and electrostatic interactions play an important role in structure and dynamics of its solutions are typically neglected.^34^ Charged polymers, in general, are known to differ from their neutral counterparts, even on a qualitative level, as attested by different concentration dependences of osmotic pressures and viscosity or different positions of crossovers between entanglement regimes above the overlap concentrations.^35−39^ However, the interplay between the ring topology and polyelectrolyte effects is understood only poorly.

Here we provide a comprehensive description of the structure, self-assembly, and dynamics of charged semiflexible mini-rings, akin to the DNA mini-rings,^40−42^ across a range of concentration to bridge the knowledge gap between the topology-driven phases of circular macromolecules and that of the DNA nanotechnology with biological and biomimetic applications. We focus on semiflexible rings, residing at the nanoscopic length scales between rigid molecular rings,^43^ such as cyclodextrins,^16^ and long flexible synthetic polymers.^44^ Such semiflexible rings possess significant intramolecular entropy and vast conformational landscapes, characteristic for polymers, allowing the neutral rings to undergo density-induced clustering, forming a fluid of anisotropic cylindrical nanoscopic stacks of mostly disk-like structures sitting on top of each other.^13,45−47^ Such systems can give rise to a cluster glass phase,^45^ in which the matrix of stacks exhibits positional arrest of the clusters, signaling an incipient glass transition. Nevertheless, on the level of a single ring in a stack, the system can relax, thereby engendering a striking decoupling between self and collective relaxation time scales. We show and explain how these phenomena are affected as we move from generic bead–spring models to the real systems of the charged rings. The like-charged mini-rings experience stronger repulsions as compared to their neutral bead–spring counterparts, which affects their assembly into nanostacks. We delineate the role of electrostatic interactions in these systems, which were shown to be relevant in a dilute single-ring case,^48^ and show how its manipulation using counterions of different valences can be used to control the morphology of the concentrated phases of such rings.

We undertake this thorough study using molecular dynamics simulations with an extension of the bead–spring model with full treatment of long-range Coulomb-like electrostatic interactions, simulating also explicit counterions in an implict solvent, as explained in detail in Methods. We use a coarse-grained model, in which we map three units of ssDNA into a single monomeric unit with a charge number of −3 and effective size of ∼1 nm, tuning the parameters to attain the persistence length and bare charge density of ssDNA.^27^ We explore a full order of magnitude of concentrations above the overlap density of the rings for three different counterion conditions: only monovalent ions, only trivalent ions, and a mixture of both as shown by the snapshot in Figure 1a. We describe and explain the structure of the emerging phases and conformations of the individual rings (Figure 1b) and identify the cylindrical clusters, shown in Figure 1c,d. We also probe the dynamics of emerging glassy fluids and explore the relaxation of rings, facilitated by threading and interpenetration.

**Figure 1:**
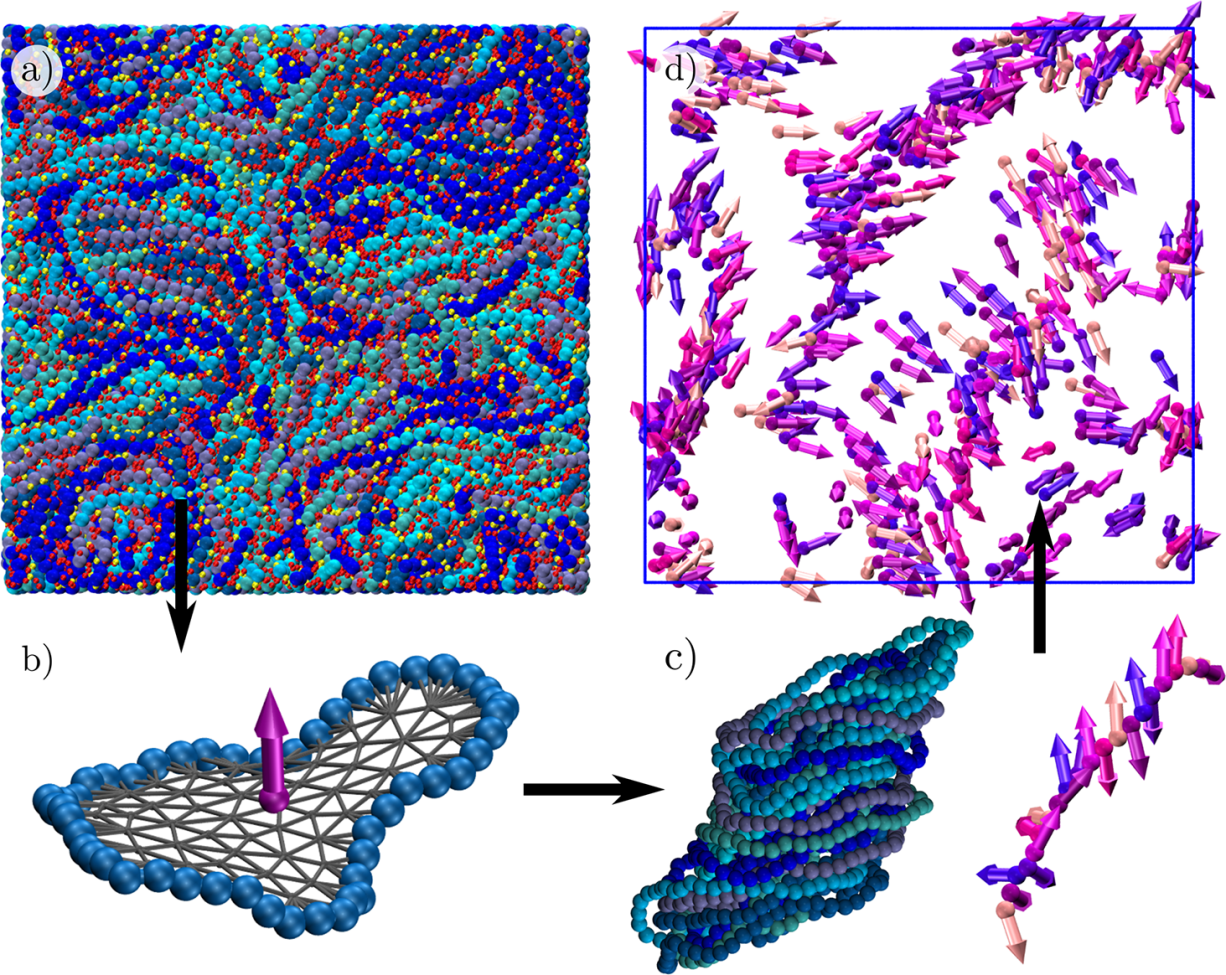
Model. (a) Snapshot of a representative configuration of a system with both monovalent (red) and trivalent (yellow) ions with polyelectrolyte rings (shades of blue) at density ρσ^3^ = 0.40. (b) Typical configuration of a single ring (blue) from the system in the top left panel with the center of mass and the director, d⃗, shown in purple and the minimal surface of the ring as gray triangles. (c) Cylindrical stack (cluster) of rings with aggregation number *N*_*s*_ = 20 taken from the system in the top left panel, shown in both monomer-resolved (shades of blue) and coarse-grained representation (shades of purple) as defined in Methods. (d) Centers of mass and directors (shades of purple) of the system in the top left panel, showing the formation of stack clusters.

## Results and Discussion

### Structure, Clustering, and Phase Separation

**Figure 2:**
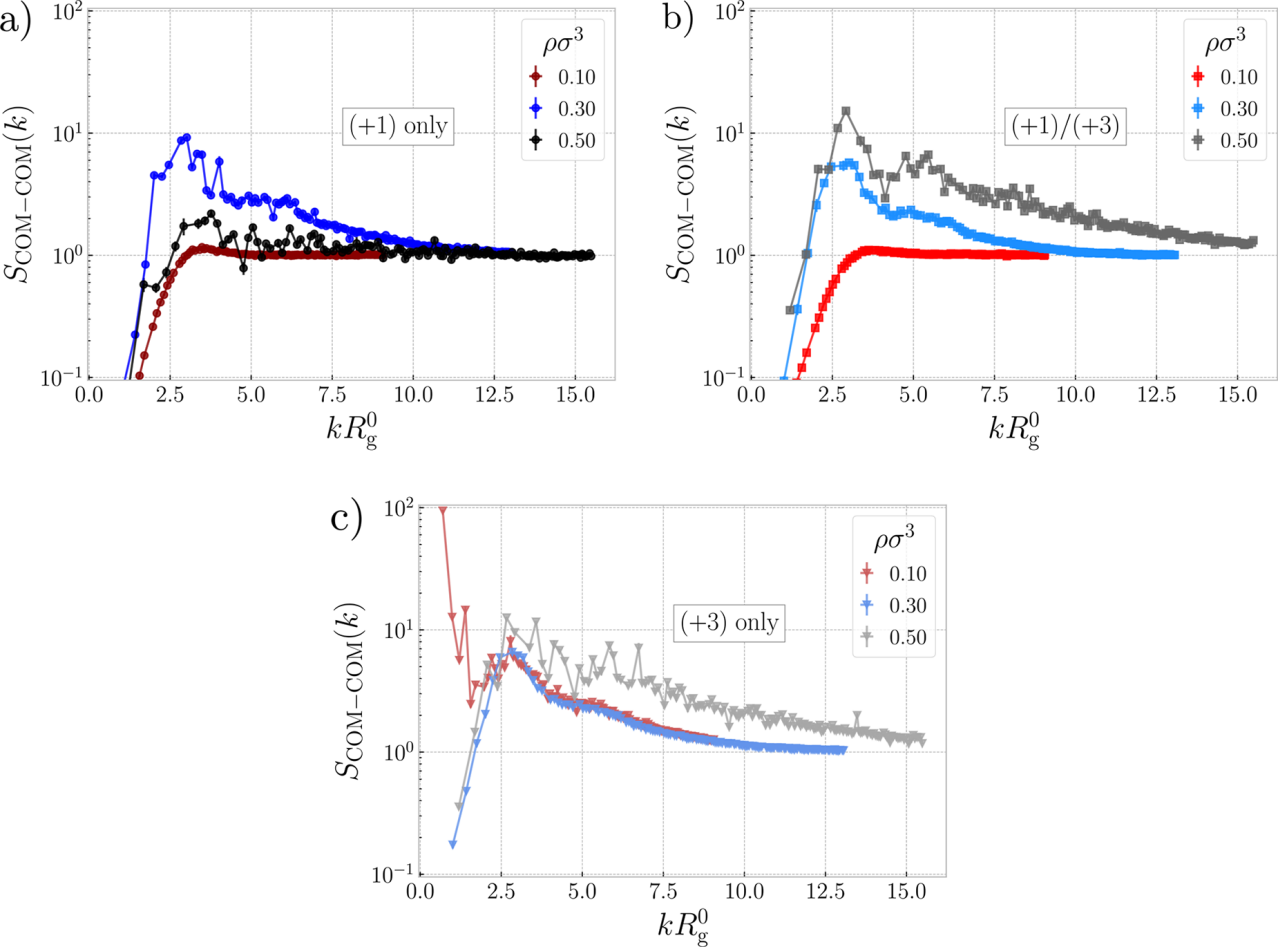
Structure factor. Static structure factor between centers of mass of the polyelectrolyte rings, for different densities and for the system with monovalent ions only in (a), both monovalent and trivalent ions in (b), and only trivalent ions in (c). The wavenumber is scaled by the radius of gyration of a single ring in infinite dilution. Analogous plots for additional values of the density are given in Section S1 in the Supporting Information.

**Figure 3:**
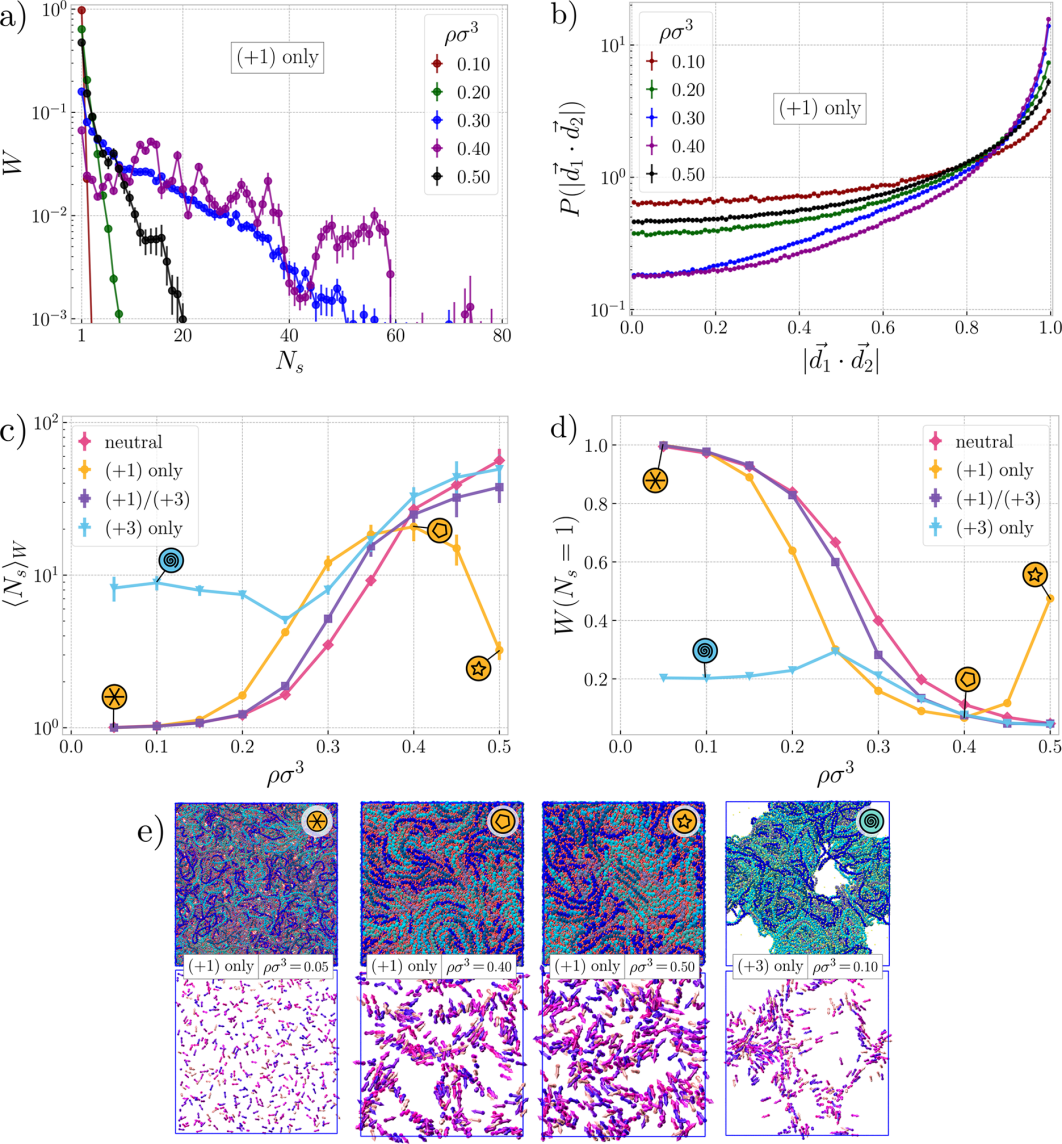
Clustering. (a) Weight fraction, *W*, and distributions of aggregation numbers, *N*_s_, of rings in the emerging cylindrical stacks for different densities for the system with monovalent ions (plots for other ion types are given in Section S1 in the Supporting Information). (b) Probability distributions of mutual orientations of directors, , or pairs of rings 1 and 2, separated by at most 0.5*R*_g,0_ plotted for different densities for the system with monovalent ions only. (c) Weight-weighed mean aggregation number, , of rings in an average stack as a function of density for different ion types, with a purely neutral system with no ions for a reference. Four bold symbols in circles mark selected systems shown in the snapshots in the bottom panel. (d) Fraction of dangling rings, *W*(*N*_s_ = 1), i.e. rings not belonging to any cluster as a function of density. (e) Snapshots of selected systems using the color code of Figure 1.

Herein, we describe the static behavior of the system as a function of density and the counterion charge using the following strategy: we delineate the global structure of the liquid phase using the structure factors (Figure 2) and simultaneously we characterize the finer structure of the emerging clusters with their weight fraction, size distributions, and director correlation of neighboring rings (Figure 3).

The structure factors in Figure 2 are calculated between the centers of mass of the individual rings, defined as
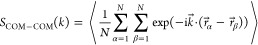
1where,  is the position vector of the center of mass of the ring α, and we average over all legal k⃗ and all configurations of the system. We consider the structure factor for all counterion types and for selected densities in the lower, middle, and upper ranges of the explored compositions.

First, the system with only monovalent ions at ρσ^3^ = 0.10 in Figure 2a exhibits a typical structure of a simple liquid of soft particles.^49^ At short separations, there is a correlation hole (as can be seen in Section S1 in the Supporting Information), caused by short-range repulsions between the rings. Contrary to the case of steep repulsions, however, the *k*-space correlation peak between nearest neighbors at *kR*_g,0_ ≈ 3.4 is weak. The overall homogeneous structure of the fluid can be seen at the far left snapshot in Figure 3e (for ρσ^3^ = 0.05). Moving to intermediate density ρσ^3^ = 0.30, we observe development of a strong correlation peak at *kR*_g,0_ ≈ 2.7, followed by a slow decay for increasing wavenumbers. The slow decay at large *k* values is a consequence of penetrations between rings, as the threading allows for centers of mass of two rings to come to arbitrarily close distances.^45^ The structural peak is not due to ring–ring correlations, since it occurs at a smaller *k* value than that for the lower density; rather, it is evidence of the onset of clustering between the rings.^50^ The clustering above at the intermediate densities is manifested by the formation of cylindrical stacks as presented in the center left panel of Figure 3e, where we show centers of mass and directors of the rings at ρσ^3^ = 0.40. In this density regime, the electrostatic self-repulsions and bending rigidity impose an energetic penalty for the crumpling of the rings, which subsequently exhibit swollen and open conformations. Stacking emerges as a way to maximize conformational entropy. It is observed also for neutral rings with no counterions,^47^ and is deeply connected to clustering of *Q*^±^-class systems,^51^ the latter being systems with bounded effective potentials, whose Fourier transform oscillates around zero.

Increasing the density further to ρσ^3^ = 0.50 leads to a decrease in clustering, as the correlation peak in Figure 2a is lost. Concomitantly, the long tail in Figure 2a fades away as the threading between rings is suppressed. Disappearance of the stacks is also qualitatively captured in the center right panel of Figure 3e, which indeed appears more homogeneous compared to ρσ^3^ = 0.40 on the center left. The nonmonotonic nature of stacking as a function of density is further confirmed by Figure 3a, where we evaluate the aggregation numbers of the emerging stacks, *N*_s_, equivalent to the sizes of connected components of connectivity matrix (explained in Methods). This weakening of the stacks is driven mainly by the osmotic collapse^52^ of the rings, as the osmotic forces of counterions act against the chain entropy, causing the rings to deform, which in turn cannot be ordered in long stacks. The loss of ordering is apparent in Figure 3b, where we show the probability distribution of the orientation of neighboring rings (separated ≤0.5*R*_g,0_). At the intermediate density, ρσ^3^ = 0.30, in Figure 3b nearby rings prefer a parallel alignment , facilitating the formation of cylindrical stacks. On the other hand, at high density, ρσ^3^ = 0.50, we see the emergence of a broad range of nonparallel orientations and the ratio of parallel-to-perpendicular probability density is ≲10 as compared to the ≳100 for the above case of ρσ^3^ = 0.30 with long stacks. Quantitatively, this weakening of stacking can be seen also in Figure 3c, showing the mass-averaged *N*_s_, calculated from the distributions in Figure 3a, and in Figure 3d, showing the population of dangling rings (*N*_*s*_ = 1) not belonging to any cluster. For both ρσ^3^ ≲ 0.20 and ρσ^3^ = 0.50, the distribution of aggregation numbers is dominated by dangling rings, then followed by small clusters *N*_s_ ≲ 10. On the other hand, the intermediate densities show broad distributions, with some stacks comprising several tens (up to ∼80) of rings.

In contrast to the systems with only monovalent ions, the monovalent and trivalent mixtures support cylindrical stacks even at high densities. The cluster correlation peak and long tail in Figure 2b is present even at ρσ^3^ = 0.50, and the position of the peak is virtually independent of the density, which is a typical characteristics of the cluster phases.^50,53,54^ The stacks keep monotonically growing with increasing density as attested by Figure 3c) (see also Section S1 in the Supporting Information). In this regime, we do not observe collapse and deformation of the rings as we do for the system with only monovalent ions. As we will show later, the trivalent ions exhibit strong counterion condensation, effectively renormalizing charge on the rings, mitigating the electrostatic repulsions between the rings, and avoiding the collapse present in the system with monovalent ions only. As a result, in the mixture of ions, the propensity for stacking increases monotonically with the density, a behavior also observed for neutral rings with no counterions. Nevertheless, the intricate balance of the conformational entropy of rings, the translational entropy of ions, and the interactions between charged species will be explained in depth in the following sections.

When comparing the monovalent/trivalent mixture to the monovalent-only case, the main differences lie in the high-density regime, while for lower densities, the two systems behave similarly. This is not the case when comparing the mono-/trivalent mixture with the trivalent-only case, as the two deviate precisely in the low-density regime while being similar in the high-density regime. For ρσ^3^ = 0.10 in Figure 2c, we see a distinctive scattering peak for the limit of *kR*_g,0_ → 0, which is a sign of a macroscopic phase separation within the system. The rightmost panel in Figure 3e indeed shows that the charged rings glued by trivalent ions form concentrated domains in the box, resembling the liquid–gas-like phase separation known in the context of polyelectrolyte complexes. Figure 3c,d shows that the mean aggregation number of stacks and number of dangling rings is almost constant in the regime 0.05 ≲ ρσ^3^ ≲ 0.20, because within this regime, the resultant phase of rings glued by trivalent counterions has almost the same local density and hence effectively corresponds to the same coexistence density. Changing the mean density of polymer in the box thereby effectively changes only the volume of the gas (almost empty) phase region. Nevertheless, for ρσ^3^ ≳ 0.25, we no longer observe the demixing and we have a stable phase of rings, as the scattering peak at *kR*_g,0_ → 0 disappears (Figure 2 and also Section S1 in the Supporting Information). Within this one phase region, increasing the density results in longer stacks and fewer dangling rings, as shown in Figure 3c,d, very similar to the cases with mono-/trivalent mixtures.

In summary, the formation of cylindrical stacks as a function of density increases monotonically for the charged rings with the mixture of mono-/trivalent counterions and survives up to high densities. If only trivalent ions are present, stacking emerges also at low densities, due to the cohesive counterion-mediated attractions between the rings, and also survives up to the high densities. If only monovalent ions are present, stacks disintegrate at high densities because of the conformational changes of the rings induced by osmotic pressure of the ions, which we explore in detail in the following section.

### Sizes and Shapes of the Rings

**Figure 4:**
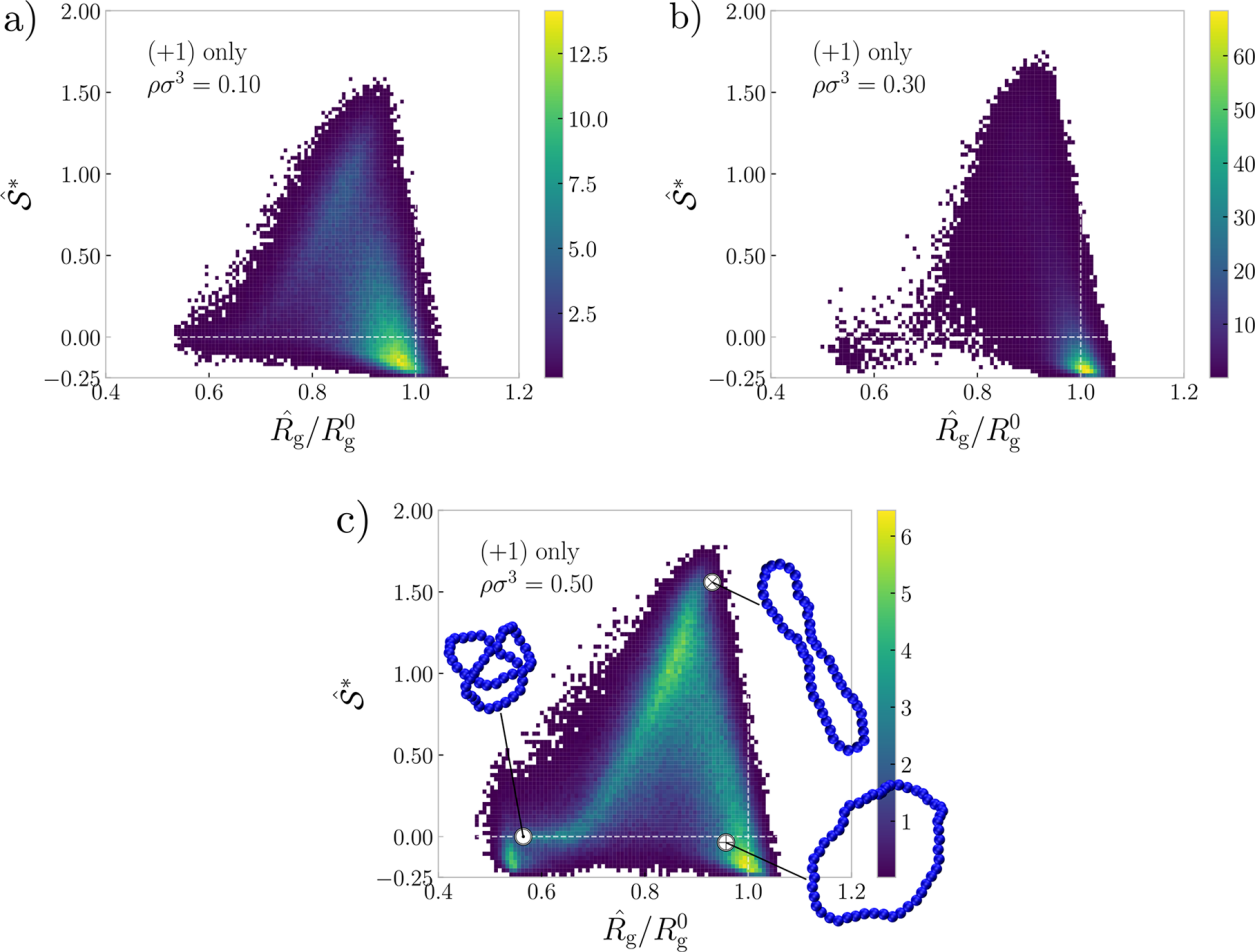
Conformations. Joint probability distributions of the instantaneous radius of gyration and instantaneous prolateness (defined in eq 10) of individual polyelectrolyte rings in the systems. All panels correspond to the systems with only monovalent ions, at densities ρσ^3^ ∈ {0.10, 0.30, and 0.50} in (a–c) respectively. The three selected points and corresponding insets show representative conformations of the ring. The radius of gyration is scaled by the radius of gyration of a single ring at an infinite dilution. Dashed white lines mark  and .

After exploring the structure of the system as a whole, we turn our attention to the single-macromolecule level. In Figure 4a we show joint probability distributions of the radius of gyration, , and prolateness, , (see Methods) of a single ring with the monovalent ions at ρσ^3^ = 0.10, corresponding to a homogeneous solution with no clusters, like the one in far left of Figure 3e (for ρσ^3^ = 0.05). The maximum for  attests to conformations being mostly disk-like and open, while elongated cigar-like configurations  or spheres  are less common than disks. This shape can be attributed to the bending stiffness of the rings, which alongside the self-repulsions of the monomeric units imposes a penalty on crumpling. Nevertheless, because of the electrostatic screening by the counterions and due to the repulsions between different rings, the macromolecules are on average slightly smaller than a single ring at the infinite dilution as most of the configurations exhibit .

At the intermediate density, ρσ^3^ = 0.30, in Figure 4b, the average ring size exceeds that at infinite dilution, which is caused by the onset of cluster formation. In the clusters, the rings strongly favor swollen disk-like shapes , which facilitates formation of the stacks, and in turn, parallelly stacked rings suppress extrusions of the subchains out of the plane of the ring, resulting in an increased radius of gyration. Concurrently, the landscape of conformations in Figure 4b is narrower compared to the case of low density, as the majority of the rings are swollen oblates, with other shapes and sizes being a minority.

Finally, at ρσ^3^ = 0.50 in Figure 4c, where the long stacks disintegrate, we see a strong heterogeneity of sizes and shapes. Their diverse landscape spans almost the full range of possible values of prolateness and ranges to as low as half of the infinite dilution radius of gyration in terms of size. We can identify three local maxima on the landscape (snapshots in Figure 4c), corresponding to the following categories. First, the open oblates, which reside in the short surviving stacks, *N*_s_ ≲ 20, are still the most probable conformations. Second, a substantial population of elongated cigar-like prolates emerges, and third, a population of shrunken double-folded rings emerges, both of them being mostly dangling rings residing outside of the stacks. As we will show in the next section, at this high density, the monovalent ions change the balance of the free energy, cause the collapse of many rings, and disrupt long stacks, breaking them into shorter ones, mitigating the electrostatic repulsions.

In contrast to the monovalent ions presented above, the systems with trivalent ions or mixtures of ions attain swollen disk-like conformations at both intermediate and high densities (Section S2 in the Supporting Information), since the osmotic collapse does not take place, due to the different nature of electrostatic interactions of different valences. For the system with only trivalent ions, counterion-mediated attractions between the rings result in stack formation at all densities, and concomitantly, rings are always swollen disks.

### Role of the Counterions and Charge

**Figure 5:**
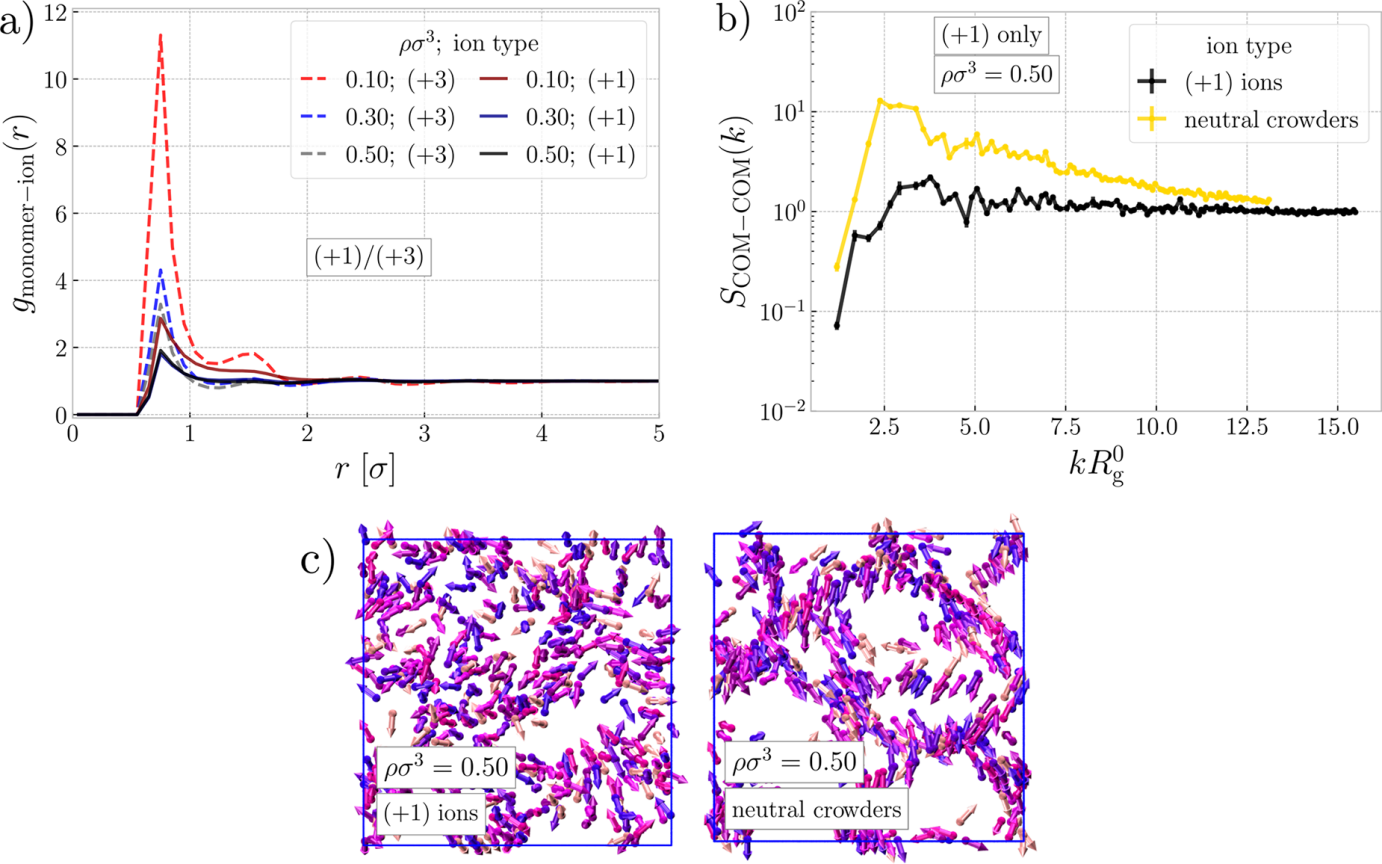
Counterions. (a) Pair correlation functions between monomeric units of rings and counterions of both types for the systems with a (+1)/(+3) mixture of ions. (b) Static structure factor between centers of mass of the polyelectrolyte rings, for the system with only monovalent ions and for the same system but with no electrostatics, thus turning ions into neutral crowders. The wavenumber is scaled by the radius of gyration of a single ring in infinite dilution. (c) Snapshots of the two systems from (b) showing recovery of the clusters, once the electrostatic is switched off. The color code is the same as in Figure 1.

To understand how different valences change the clustering behavior, it is instructive to look at the systems with a mixture of both monovalent and trivalent ions. In Figure 5a, we show pair correlation functions between charged monomers and the counterions. Across all densities, the trivalent counterions show a strong correlation peak at ∼0.7 nm ≈ (1.0 nm + 0.355 nm)/2 roughly equal to the sum of radii of the ring monomeric unit and the ions. This indicates a strong localization of trivalent ions in the very close vicinity of the polymer, which is also supported by the presence of a secondary correlation peak in *g*(*r*) at ∼1.4 nm, corresponding to the distance of the ion from the next monomer along the contour. On the other hand, the monovalent ions show more homogeneous profiles with a less pronounced structure and, even though they also accumulate around the rings, their affinity for the charged polymer is much weaker when compared to their trivalent counterparts. These effects are well-known in the field of polyelectrolytes in the context of counterion condensation.^55−58^ To make a connection to the theory of condensation, the contour charge density of our rings is ∼3*e*/nm ≈ 2.1*e*/*l*_B_, resulting in the Manning parameter ∼2 for monovalent or ∼6 for trivalent ions, both of which are above the critical value of unity. Our simulations show that in the systems with a mixture of ions, effectively all of the trivalent counterions are condensed on the chain, while the cloud of monovalent ions is spread around the voids in the system (visible in Figure 1a). The different propensities for the counterion condensation for the two valences is the main source of disparities between the systems.

We claim that the dissolution of long stacks at ρσ^3^ = 0.50 in the systems with only monovalent ions is caused by the above interactions of the ions. For the monovalent system, only a fraction of counterions will condense, renormalizing the Manning parameter to unity, while the remainder of the ions is unbound and occupies mainly cavities in the system. However, if such a system contained long cylindrical stacks, most of the cloud of free ions would have to occupy the free space inside of the cylinders. Such a conformation would exhibit strong electrostatic repulsions—first, between the highly charged rings sitting right on the top of each other and, second, between the free ions, all localized inside and between the stacks. The above segregation of charge would create a strong free energy penalty, breaking the long stacks into smaller ones, separated by individual collapsed rings of different shapes helps to alleviate the electrostatic repulsions at the expense of bending energy of the rings and loss of entropy of stacks.

To support this assessment, we explored an auxiliary reference system with no charging (Figure 5b,c). We took the system with monovalent ions at ρσ^3^ = 0.50 (depicted on the left part of Figure 5c) and switched off the electrostatic interactions, hence turning the charged rings into neutral rings and the counterions into the neutral crowders. After equilibration and simulation of this system, long stacks were formed as shown in the right panel of Figure 5c and also in the structure factors in Figure 5b, where the correlation peak and long tail are recovered. Therefore, the equilibrium structure of the neutral system unambiguously demonstrates the essential role of the electrostatic interactions in the disappearance of long stacks and collapse of some of the rings in the monovalent system at high densities. Interestingly, this behavior is observed only when using the explicit long-range Coulombic potential with explicit ions. Instead, using the screened (Debye–Hückel) interaction potential matching the ionic strength of the explicit system did not capture the collapse of the rings (Section S3 in the Supporting Information).

Finally, in the system with the mixture of monovalent and trivalent ions, the stacks keep growing monotonically with the density, and we do not observe their breakdown at ρσ^3^ = 0.50. As noted above, in this system essentially all of the trivalent ions strongly condense on the rings, effectively decreasing the contour charge density twofold. Concurrently, the number of (free) monovalent ions in this system is lower as compared to the case of only monovalent ions. As a result of these two effects, the rings in stacks and ions in the voids both experience fewer repulsions as compared to the case with only monovalent ions and the free energy penalty is not strong enough to break the stacks and deform the rings. In a sense, a small addition of trivalent ions effectively shifts the systems from the high-charge-density limit to the direction of the weak-charge-density limit, closer to the neutral systems. Accordingly, we would expect that stacks might actually survive even without trivalent ions, providing that the bare charge density of the rings is low enough or permittivity high enough.

### Cluster Glass Dynamics

After exploring the structure of the systems, we now turn our attention to the dynamics. We first define the coherent, *F*_coh_, and the incoherent, *F*_inc_, intermediate scattering functions (ISFs)^45^
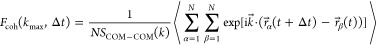
2
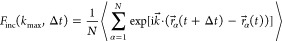
3where Δ*t* is the lag time, α and β are indices of the rings, and the averaging is done over different time origins and over all allowed  vectors, with the magnitude, *k*_max_, corresponding to the maximum of the density-independent scattering peak in Figure 2. The former of the two functions probes the collective relaxation of the system, while the latter probes the self-relaxation of the individual rings.

**Figure 6:**
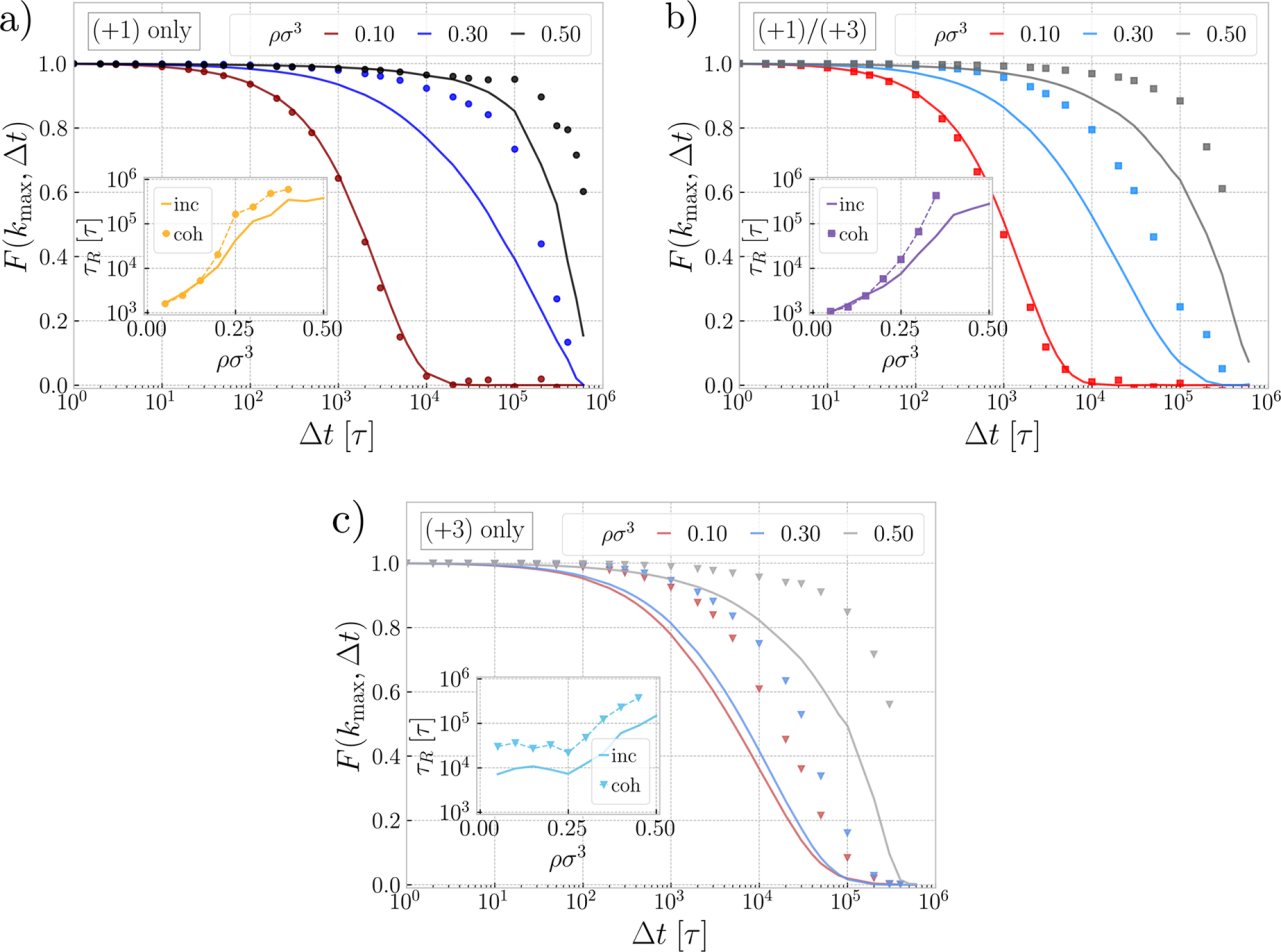
Dynamics. Intermediate scattering functions (eqs 2 and 3) for centers of mass of the polyelectrolyte rings compared for different densities for the system with only monovalent ions in (a), both, monovalent and trivalent ions in (b), and only trivalent ions in (c). The incoherent (self) part is plotted with lines, while the coherent (collective) part is plotted with points and *k*_max_ is the wavenumber corresponding to the main clustering peak in Figure 2. Insets show the characteristic relaxation times, τ_R_, defined as *F*(*k*_max_, τ_R_) = *e*^–1^ for both functions, respectively. Analogous plots for more densities are shown in Section S4 in the Supporting Information.

First, in Figure 6a we show the intermediate scattering functions for the system with only monovalent ions. For the solutions at low density (ρσ^3^ = 0.10) with virtually no clustering, the collective part is the same as the self-part, and in the limit of a long time, the rings reach Brownian diffusion, as expected for simple liquids. At the intermediate densities (ρσ^3^ = 0.30) with onset of clustering, both collective and self-dynamics get slower; however, we see an emerging separation between the two, with the collective relaxation being the slower one. At this density, the long stacks of rings are caged by each other, and on the scale of whole stacks, the positions of clusters are getting arrested, which is a sign of an incipient glass transition. On the other hand, at the level of single rings, the individual macromolecules can detach from the clusters and hop between different clusters but can also exist as individual dangling rings, all of which is reflected by faster self-relaxation, as compared to the slow, collective one. In the inset of Figure 6a, we extract the characteristic relaxation times, τ_R_, here defined as the times where *F*(*k*_max_, τ_R_) = *e*^–1^ for both coherent and incoherent scattering functions, respectively. The striking decoupling of the two relaxation times is a characteristic feature of cluster glasses,^45^ which was originally observed for neutral rings (no electrostatics or counterions), for which the separation between the two times increase with density. In contrast, for the charged rings in the current study, we see a reversal of this trend at high densities (ρσ^3^ = 0.50), as the two scattering functions in Figure 6a approach each other, resulting in comparable self- and collective relaxation time scales. At the same time, we see a slowdown in correlations of both collective and self-motions, reflecting the arrest of correlations not only of the surviving small clusters but also of the individual rings. We note that for the densest system, we could not sample the collective correlation functions fully and thereby the accurate reading of the relaxation time is missing for some of the systems. Nevertheless, unlike the rings at intermediate densities, the self-relaxation at high densities is subordinated to the relaxation of the caging matrix, which is a signature of simple glasses. Interestingly, the self-intermediate scattering function is changing form to almost logarithmic as a function of the density around the point ρσ^3^ = 0.30 (see Section S4 in the Supporting Information) reminiscent of a higher order singularity in the glass transition.^59^ Consequently, upon increasing the density, the system exhibits behavior akin to a glass-to-glass transition, from a cluster glass of long stacks of rings to a simple glass of short stacks and individual rings. Whether there exists an intermediate fluid phase remains to be explored in the future. Note, however, that the equilibration time is on the very limit for this system (see Methods); therefore, we refrain from making definite conclusions on these points.

In line with the structural observation from the preceding sections, the system with the mixture of ions in Figure 6b shows no stacks or glassiness at a low density (ρσ^3^ = 0.10). At intermediate densities (ρσ^3^ = 0.30), the cluster glass phase emerges, as attested by the decoupling of the self- and collective time scales visible in the inset of Figure 6b. In contrast to the transition of a cluster glass to a simple glass or fluid for monovalent ions above, for the mixture of ions, we observe only the incipient cluster glasses, as the separation between the two relaxation times only increases. The long stacks survive, and accordingly, rings can relax faster than the whole matrix of stacks, due to the interstack hopping, exchange with other rings within the stack, etc. Similarly, in the systems with only trivalent ions in Figure 6c, the stacks are present at all explored densities, due to the bridging by multivalent ions and the emergent phase separation. Accordingly, cluster glass dynamics with the separated time scales are observed at all densities.

### Threading Entanglements

Further insight into the dynamics of the presented systems can be gained by exploring the threading entanglements between the rings. The swollen oblate-like rings offer a large opening, which can be easily penetrated by other polymers. As defined in Methods, we denote such an event as threading, and we would coin the penetrated ring as a passive threading partner to the active threading partner: the ring which is penetrating the opening of the other. In contrast to the oblate-like rings, the elongated prolate-like rings possess rather tight openings, while at the same time, their rod-like shape makes it easy to pierce other rings, thereby turning the prolates into potential active threading partners. Threading presents a type of entanglement between polymers that is specific to rings, affecting their relaxation by entrainment.

**Figure 7:**
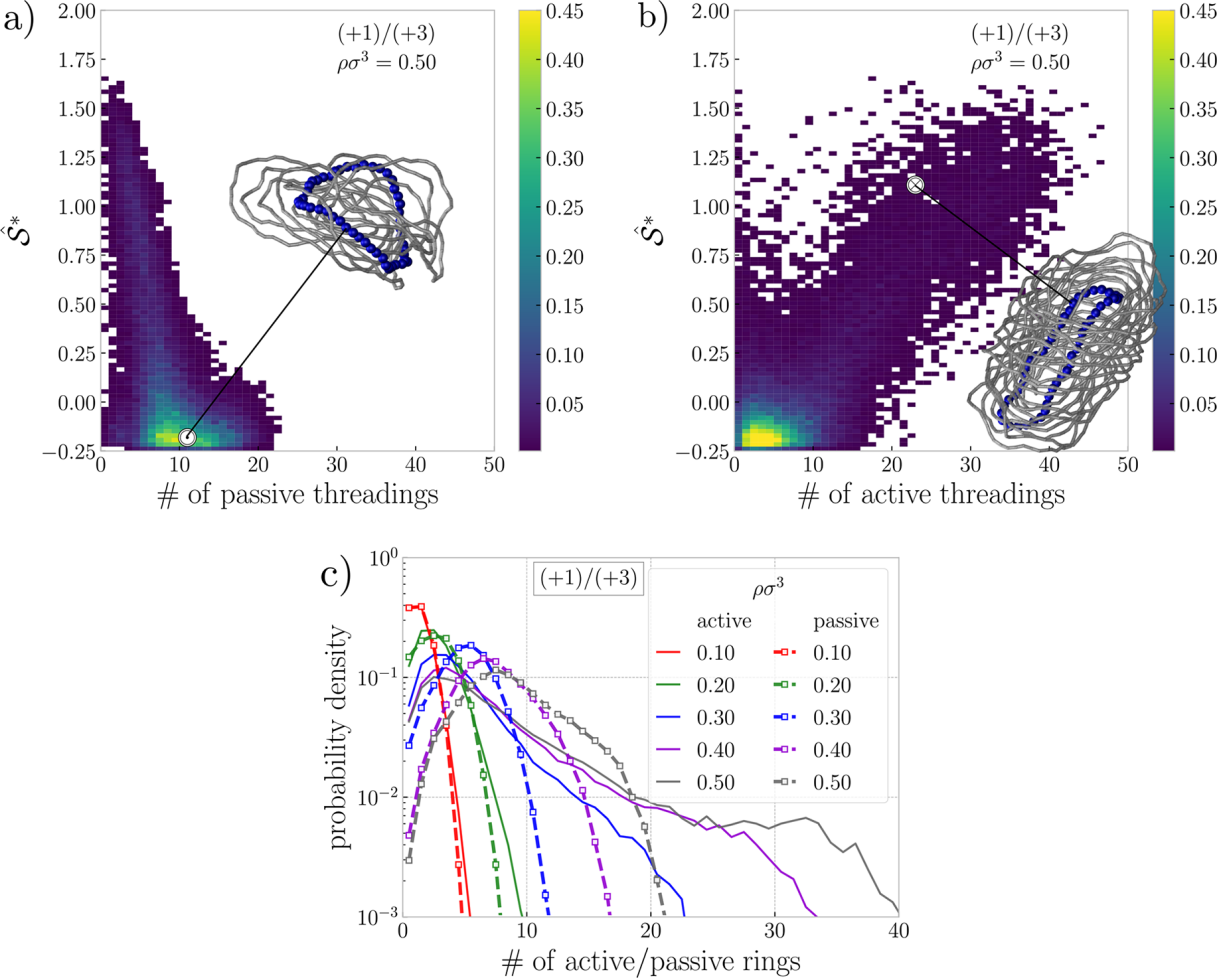
Threading. (a) Joint probability distribution correlating the prolateness of a ring (defined in eq 10) with the number of passive threadings (how many other rings are threading this ring). Pinpointed is the data point for the blue oblate ring threaded by the gray rings. (b) Joint probability distribution correlating the prolateness of a ring with the number of active threadings (how many other rings are threaded by this ring). Pinpointed is the data point for the blue prolated ring threading the other gray rings. (c) Probability distributions of the number of passive partners (dashed line with points) and active partners (solid lines) per ring, plotted for different densities for the system with both monovalent and trivalent ions. Analogous plots for additional systems are given in Section S5 in the Supporting Information.

Following the protocol described in Methods, for each ring, we count how many partners are threading it and also how many other rings are being threaded by it. In Figure 7a we correlate the distribution of numbers of the passive (received) threadings per ring with the shape of the ring, for the system with mixed ions at the highest density ρσ^3^ = 0.50, the system where the long stacks survive. First, the most typical ring is an oblate  threaded by ∼10 other rings as exemplified in the inset of Figure 7a. Possible numbers of the passive threadings of an oblate ring range from 0 up to ∼20, where the upper limit roughly corresponds to the ratio of ring density to overlap density. Contrarily, cigar-like prolates  are threaded by others significantly less as compared to the oblates, since their opening has a small area for penetration.

In Figure 7b, we show an analogous analysis, correlating the shape of the rings with the number of other rings getting pierced by it (number of active threadings per ring). A typical oblate is threading only ∼5 other rings, while prolates can thread up to ≲50 other rings in line with the above discussion. The inset of Figure 7b shows a representative conformation of a prolate, nesting inside a cylindrical stack and threading many other rings, all of which are part of the same cluster. This, however, reveals that in the cylindrical stacks the rings are not perfectly aligned but rather are tilted with a lot of interpenetration, as is also visible in the inset of Figure 7a, where all the rings in the snapshot are a subset of a single stack.

The existence of long prolates stabilizes the long stacks and also contributes to the dynamic arrest of the positions of the stacks and the emergent cluster glass formation. Due to the central prolate in a stack, the motions of threaded oblates are laterally restricted, and we suppose they diffuse mainly along the axis of the stack, while they can exit the stack only at the ends. In turn, the diffusion along the axis of the stack is perpetuated by threading between the oblates (inset of Figure 7a), which can pass each other, hence allowing relaxation of a single ring, while the stacks are being arrested. In Figure 7c we present the threading relations as a function of density, showing that strongly threading prolates (the long tail for ≳20) occur precisely in the systems with long clusters. For the low densities without the cluster phase, the distributions for both active and passive threading are almost the same, as expected.

In general, there is a slight preference for shallow threadings (Figure S10 in the Supporting Information) in an otherwise broad landscape of threading depths. The only major deviation from this trend is visible in the collapsed phase with monovalent ions at ρσ^3^ = 0.50 (Figures S10 and S11 in the Supporting Information). In the phase of collapsed rings, deep threadings are very rare, and most of the threadings are just a few monomeric units deep. In this phase, the surviving stacks are short, and the dangling rings shaped as prolates and double-folded globules have a rather closed opening, both of which disfavor threadings. In concentrated systems of neutral rings, we typically see that with increasing density we have more threading. In the melts of long rings the threading entanglements are suspected to cause a slowdown of the system.^60,61^ In the present systems of shorter rings, we see that threading has only a limited impact on the ring relaxation. The systems in their cluster phases (ρσ^3^ = 0.30) are similarly threaded with similar relaxation (monovalent slightly slower), while at higher density, ρσ^3^ = 0.50, the monovalent collapsed phase has less threading than other systems, but does not exhibit faster relaxation (see Section S4 in the Supporting Information).

## Conclusions

We used molecular simulations to probe the structure and dynamics of concentrated solutions of semiflexible polyelectrolytes in the presence of monovalent counterions, trivalent counterions, and also the mixture of ions of both valences. Clustering of rings in cylindrical stacks is a generic feature in the explored systems; however, counterions can steer the morphology and dynamics of the stacks.

If only monovalent ions are present, the rings exhibit a nonmonotonic propensity for stacking as a function of the polymer concentration. The clusters present at intermediate densities disintegrate at high densities, due to the collapse of individual rings, disfavoring formation of long stacks. In the collapsed phase, the rings attain conformations in a broad range of sizes and shapes ranging from double-folded globules to elongated prolates and swollen disk-like oblates, in contrast to the rings in the cluster phase where the disk-like oblates vastly dominate. We attribute the collapse to the electrostatic interactions in the system, since the monovalent counterions do not exhibit strong Manning condensation and are mostly delocalized between and inside of the stacks. The formation of long stacks with such a distribution of ions would lead to strong charge segregation and electrostatic repulsions; thereby, the collapsed phase with short stacks and collapsed rings is preferred. The above statements are supported by the auxiliary simulations, in which we switched off the electrostatics in the collapsed phase, hence turning the polyelectrolyte rings into neutral ones and counterions into neutral crowders. Without electrostatics, long stacks were formed, and conversely, the emerging long stacks of neutral rings disintegrate anew upon switching the electrostatics on again.

In the phase where long stacks are present, the systems exhibit characteristic cluster glass dynamics with the decoupling of self- and collective relaxations. While the positions of stacks show positional arrest, the individual rings can relax on a faster time scale. This relaxation is facilitated by the internal structure of the stacks, where the rings are typically shallowly threaded to each other, being penetrated. Since the rings have an open interior, they can pass each other, slip through, and move along the axis of the stacks, a dynamic model which would not be accessible to colloidal disks or other impenetrable objects. Additionally, we observed the rings at the boundary of the stacks to detach and dangle, sometimes even hopping to a different cluster. In the dense collapsed phase, however, the system resembles a simple glass, as the self- and collective relaxations have comparable time scales, while both show an arrest of correlations. The polyelectrolyte rings thereby exhibit a behavior akin to an incipient glass-to-glass transition, where the incipient glass of stacks is turned into an incipient glass of rings, upon increasing the density.

In contrast to the monovalent ions, the trivalent ions show a strong counterion condensation, effectively reducing and renormalizing the charge density of the polymer. Accordingly, upon a small addition of trivalent ions, the propensity for stacking is greater than for the systems with only monovalent ions. In such systems, we also do not observe any collapse of the rings at high densities, and the system exhibits cluster glass but not simple glass dynamics. If the addition of multivalent ions is stoichiometric, then the ions can drive the phase separation of the system, forming a polyelectrolyte-ion complex and further stabilizing the clusters by attractive bridging.

Our simulation model is a generic one, foregoing chemical details of the studied systems on purpose, which underlines the universality of the results as long as the ring length remains comparable to the persistence length (*N*σ ≃ 8*l*_per_) and therefore the conformational entropy of the ring is limited, leading to ring stacking, not observed for fully flexible rings. A further increase of the stiffness might lead to ordered columnar phases, while the study of mixtures of rings with different flexibilities and/or lengths would be very interesting. There we expect a rich behavior as the stacking of the stiffer rings, complemented by active threading of the more flexible ones,^14,62^ competes with the counterion effects. Nevertheless, the presently studied conditions of charge density, polymer stiffness, and mass concentration of the rings are well within the usual ranges used in DNA nanotechnology. Experimental evidence for the formation of stacks or a glass-to-glass transition is missing at the moment, but work on similar systems with DNA rings of diameter ≲10 nm is currently under way.

## Methods

### Microscopic Model

Our system contains *M* = 512 unknotted, nonconcatenated ring polymers, represented by a standard bead–spring coarse-grained polymer model.^63^ A single ring is composed of *N* = 50 monomeric units, modeled as point particles interacting with repulsive nonbonded Weeks–Chandler–Andersen (WCA) given by
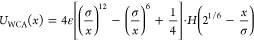
4where *x* is the instantaneous interparticle distance, ε = *k*_B_*T*, *H*(·) is the Heavside step function, and we set the length scale for the monomeric unit as σ = σ_mon_ = 1 nm ≈ 3 nt (nucleotides); hence one macromolecule corresponds to a single ssDNA ring of ∼150 nt. Monomeric units are connected by finitely extensible nonlinear elastic (FENE) bonds defined by the potential
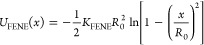
5where, *K*_FENE_ = 30*k*_B_*T*/σ^2^ and *R*_0_ = 1.5σ, which in combination with the above WCA potential yields uncrossable bonds of mean lengths ⟨*b*⟩ ≈ 0.96σ. Next, we emulate the intramolecular stiffness with harmonic cosine potential
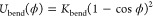
6where *K*_bend_ = 30*k*_B_*T* is the bending spring constant and ϕ is the instantaneous angle between consecutive bond vectors. In ref (14) we have shown that the persistence length of our model is roughly *l*_per_ ≈ 6σ ≈ 6 nm, which resides at the upper limit of typical persistence lengths, 0.7 nm ≲ *l*_per_ ≲ 6 nm reported for ssDNA,^64^ in strong dependence on the conditions.

Since a single polymer bead represents three nucleotides, it carries an electric charge (*q*), of charge number *z* = −3 (therefore *q* = −3*e*, where *e* is the elementary charge) to match the bare charge density of the ssDNA.^65^ To preserve the electroneutrality of the system, the total charge on the polymer is balanced by free and small monovalent (*z* = +1) or trivalent (*z* = +3) counterions. Specifically, we explore three different setups: (i) a system containing only monovalent counterions (whose count is accordingly 3*NM*), (ii) a system containing only trivalent counterions (whose count is *NM*), and (iii) a system containing both monovalent and trivalent ions, while keeping the total charge of the species the same (therefore, the system contains 3*NM*/2 monovalent counterions and *NM*/2 trivalent counterions). While small trivalent ions are rather rare in biological environments, they are commonly used in experiments on synthetic polyelectrolytes.^58,66^ From the perspective of DNA, our study of systems with trivalent ions can be regarded as an exploration of the extreme case of high valency. The counterions are modeled as explicit particles interacting with WCA potential from eq 5, but with their respective size σ_ion_ = 0.355σ = 0.355 nm, since it has been shown that this effective size reproduces well the excess chemical potentials of small ions up to the solubility limit.^67,68^ For the excluded-volume interactions between the polymer and ions, we use Lorentz–Berthelot mixing rules; hence, we adjust interaction length scale σ in eq 4 as (σ_mon_ + σ_ion_)/2. All of the charged particles participate in the electrostatic interactions, modeled with the explicit Coulomb interaction
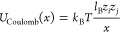
7where *l*_B_ = *e*^2^/4*πε*_0_ε_*r*_*k*_B_*T* = 0.71 nm is the Bjerrum length of the implicit solvent (dielectric continuum), corresponding to aqueous solutions at room temperature. The electrostatic potential and forces are evaluated using a particle–particle particle-mesh (PPPM) method,^69^ where we require the relative error^70,71^ of the method to be ≤10^–3^.

The charged rings with counterions are placed in a cubic simulation box with periodic boundary conditions. The size of the boxes ranges from 80σ to 37σ, covering a range of 0.05 ≲ ρσ^3^ ≲ 0.5, where ρ is the reduced particle density of monomeric units. The above range translates to roughly 2 ≲ *c*/c* ≲ 20 in terms of ring number density relative to the overlap density for the rings, or 2.5 vol % ≲ ϕ_mon_ ≲ 25 vol % in terms of monomer volume percent, or 75 mg/mL ≲ *c*_*m*_ ≲ 750 mg/mL in terms of mass concentration, where the lower bound is of the same order of magnitude as DNA in bacteria (∼20 mg/mL) or dsDNA in kinetoplasts^72^ (∼50 mg/mL), whereas the upper bound is close to the conditions in a T4 bacteriophage (∼800 mg/mL).^73^ A typical configuration of a system around the upper limit of the densities is shown in Figure 1a).

### Simulation Method

To sample the configurations of the above model, we use Langevin dynamics as implemented in LAMMPS.^74^ The governing equations of motion for each of the particles are
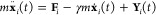
8where γ is the friction coefficient and **Y** is the random force obeying ⟨**Y**_*i*_^α^(*t*)⟩ = 0 and ⟨**Y**_*i*_^α^(*t*)**Y**_*j*_^β^(*t*′)⟩ = 2*γm*_*i*_*k*_B_*T*δ_*ij*_δ_*αβ*_δ(*t* – *t*′), where α, β ∈ {*x*, *y*, *z*} and δ is the Kronecker delta. Finally, the force **F**_*i*_ is the deterministic force originating from the gradient of potentials (eqs 4–7.) The mass of each particle is *m* = 1, which sets the time unit as , where δ*t* ≡ 0.005τ is our integration time step.

We first initialize the rings on a primitive cubic lattice in a box of size *L*_0_, guaranteeing that the rings do not possess any links or knots. Next, we run the simulation for 5 × 10^3^τ, which is of the same order of magnitude as the Rouse time of a chain with *N* = 50 monomers. Then, we rescale the coordinates of the box as *L*_0_ → *L*_0_ – σ, and we rescale the coordinates of the particles by a factor of 1 – (σ/*L*_0_), after which we run the dynamics for another 5 × 10^3^τ. We repeat the above steps until we reach the final box size, corresponding to our desired concentration. Afterward, we retune the parameters of the PPPM method and then equilibrate the system for 3 × 10^5^τ, followed up by a production simulation of length 10^6^τ. Although the equilibration time is long enough for the majority of the systems, the monovalent and stoichiometric systems at ρσ^3^ = 0.50 might be relaxed only partially (intermediate scattering functions do not reach zero, albeit the rings diffused more than their size), and therefore, the results on the dynamics should be interpreted with care.

### Directors and Identification of Clusters

To characterize the size, orientation, and conformation of the ring polymers, we use the collective variables employed also in our previous studies of neutral rings.^14^ For an instantaneous conformation of a ring, we calculate the gyration tensor, **Ĝ**, with matrix elements
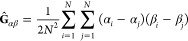
9where α, β ∈ {*x*, *y*, *z*} are the Cartesian coordinates of particles indexed by *i* and *j*. The eigenvectors of the gyration tensor are connected to the principal axes of the macromolecule, and their associated eigenvalues  relate to the instantaneous radius of gyration as  (and to its ensemble average ). Nevertheless, our rings are rather short and stiff and typically exhibit open planar-like conformations, and the trace of the gyration tensor gives no information about the orientation of such a configuration. For this reason, we define the *director*(13) of a ring as a unit vector, d⃗, in the direction of the eigenvector coupled to the smallest eigenvalue, . For a perfect circle, the director is perpendicular to the plane of the circle. Analogously, for typical conformations of short semiflexible rings, the director points in the direction perpendicular to the ring surface, as shown in Figure 1b.

To characterize the shape of rings, we use the prolateness, defined for an instantaneous configuration of a ring as
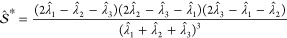
10

Generally, the prolateness shows positive values for elongated cigar-like objects (prolates), negative values for lentil-like disks (oblates), and zero for objects with spherical symmetry.

Finally, to identify and describe the emerging structure of cylindrical stacks of rings, we use the algorithm employed in our previous publications.^75,76^ We consider rings *i* and *j* to be stacked together, if the following three conditions are simultaneously fulfilled
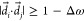
11
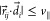
12
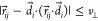
13where  is the director of the ring *i* and  is the vector between the centers of mass of the two rings. The condition of eq 11 requires the directors of the rings to be aligned in a close-to-parallel way, where we use Δω = 0.1. The conditions of eqs 12 and 13 control the distance between the rings, projected into the two directions, respectively, parallel and perpendicular to the one of the directors, where we use *v*_∥_ = 3.0σ and *v*_⊥_ = 2.5σ. As noted in ref (76), the values of the thresholds are arbitrary, but the resulting trends are rather robust to the variation of the parameters. In an instantaneous configuration of the system, we first locate all of the centers of mass of the rings and find the directors (as depicted in Figure 1c. Next, we construct a pairwise adjacency matrix,  of size *N* × *N*. Due to the underlying symmetry *ij* ↔ *ji*, without loss of generality, we construct the matrix as a strictly upper triangular one, hence  for all *i* ≤ *j*. Afterward, for each pair of the rings *i* > *j* we set  if the pair of the rings fulfills the three criteria above, or  otherwise. This matrix in turn defines a nonoriented connectivity graph of the system, in which we locate all maximal connected components, each of them corresponding to a single stack cluster.

### Minimal Surfaces and Threading Analysis

To probe the threading entanglements in the system, we first construct the minimal surface^60,61,77^ contained within the contour of each of the rings, as depicted in Figure 1b. For a given conformation of a ring, we initialize the surface as a triangular mesh spanned on vertices of two types. First, the vertices coinciding with the monomeric units of the ring are fixed. Second, additional ghost particles (gray in Figure 1b) are iteratively evolved under the imposed virtual surface tension using the Surface Evolver package.^78,79^ The minimization procedure converges if the area of the surface does not change by more than 0.1% over a period of 240 propagation steps. For more detailed description of the methods, we refer the reader to the original article^60^ and to the Supporting Information of our recent work,^14^ where we used the same parameters of the minimization procedure.

If any of the bond vectors of ring *A* intersects the internal area of any of the triangles of ring *B*, then ring *A* is threading ring *B*. In the case of such an event, we denote the ring *A* of the two rings as the active partner, while the ring *B* is the passive partner. The minimal surface of the passive partner cuts the contour of the active partner into segments, whose monomer counts we designate as threading depths, {*L*_t_}, characterizing the degree of penetration of the threading.

## Supporting Information Available

Additional plots showing structure factors, radial distribution functions, threading analysis, intermediate scattering functions, radii of gyration, and prolateness for all of the investigated systems (PDF)
