# Supplementary material for: Cluster Formation in Solutions of Polyelectrolyte Rings
Source: ACS Nano. 2023 Sep 20;17(21):21369–82. doi: 10.1021/acsnano.3c06083 (PMC10655244; doi:10.1021/acsnano.3c06083)
Supplement: Supplementary file 1 — nn3c06083_si_001.pdf [file nn3c06083_si_001.pdf]

Supporting Information: Cluster Formation in Solutions of Polyelectrolyte Rings

Roman Staňo,[*,†,‡] Ján Smrek,[†] and Christos N. Likos[†]

†*Faculty of Physics, University of Vienna, Boltzmanngasse 5, 1090 Vienna, Austria*

‡*Vienna Doctoral School in Physics, University of Vienna, Boltzmanngasse 5, 1090 Vienna, Austria*

E-mail: roman.stano@univie.ac.at

S1 Structure

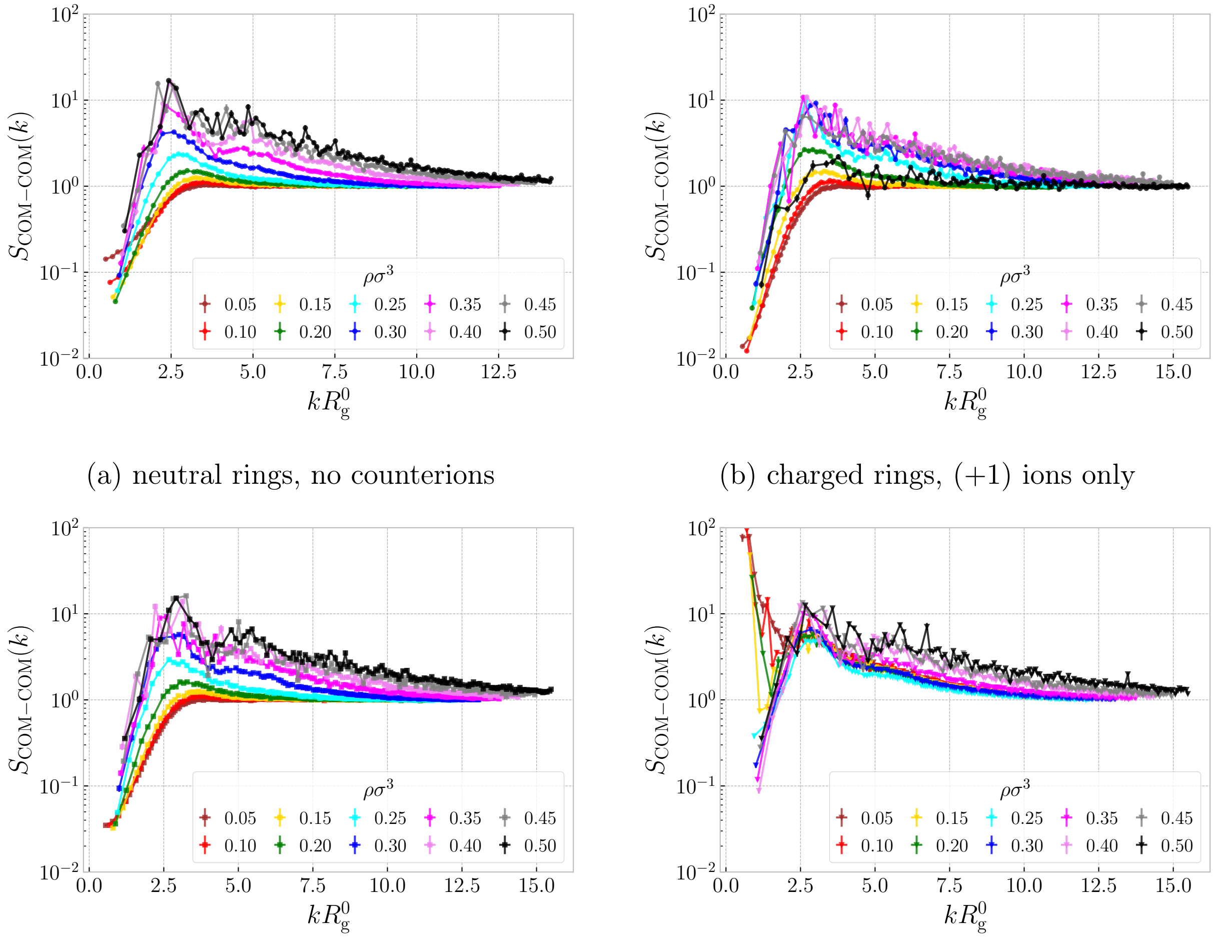

(a) neutral rings, no counterions

(b) charged rings, (+1) ions only

(c) charged rings, (+1)/(+3) ions

(d) charged rings, (+3) ions only

Figure S1: Static structure factor between centers of mass of the rings for different densities and counterion types. The wavenumber is normalized by the radius of gyration of a single ring in infinite dilution.

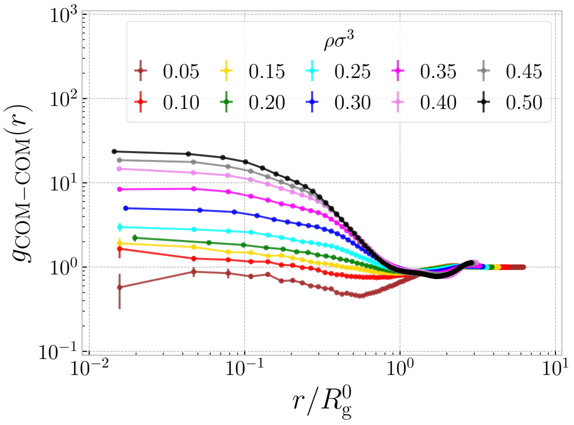

(a) neutral rings, no counterions

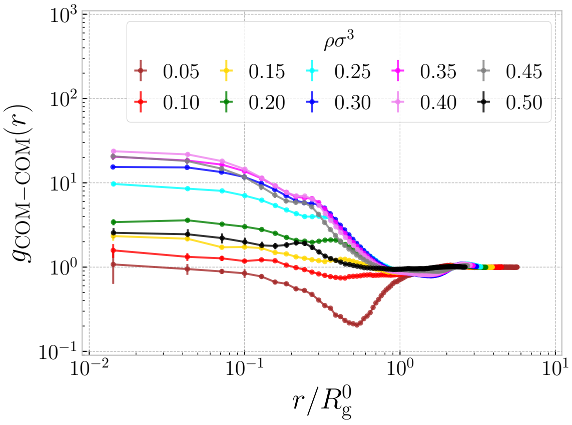

(b) charged rings, (+1) ions only

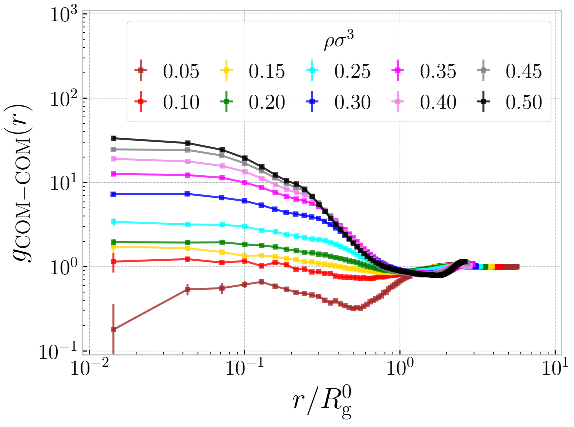

(c) charged rings, (+1)/(+3) ions

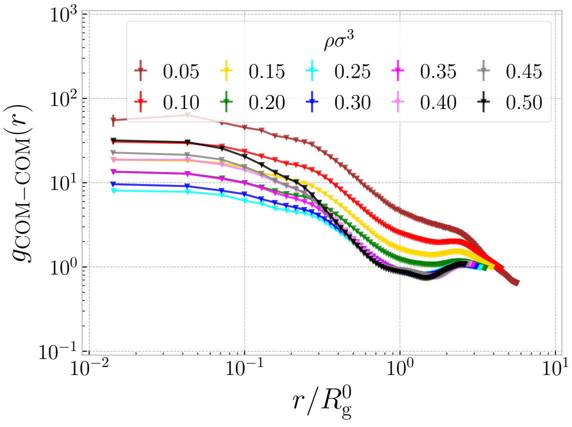

(d) charged rings, (+3) ions only

Figure S2: Radial distribuiion functions between centers of mass of the rings for different densities and counterion types. The distance is normalized by the radius of gyration of a single ring in infinite dilution.

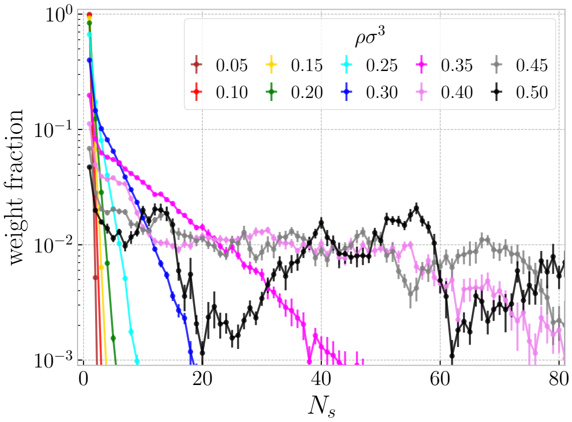

(a) neutral rings, no counterions

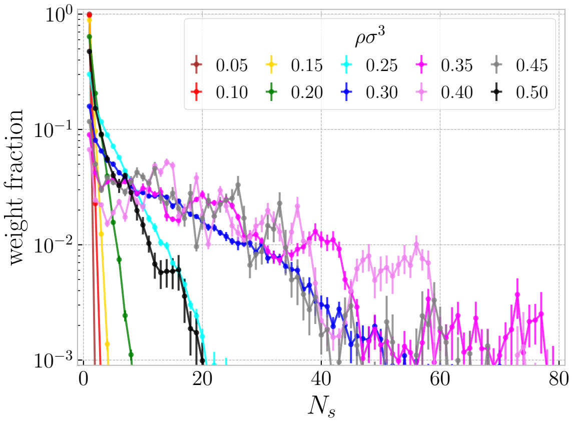

(b) charged rings, (+1) ions only

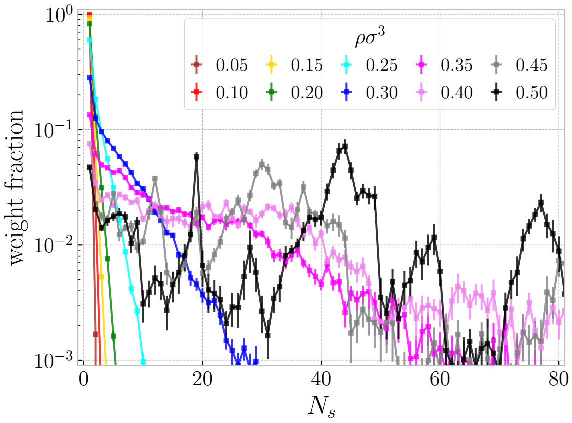

(c) charged rings, (+1)/(+3) ions

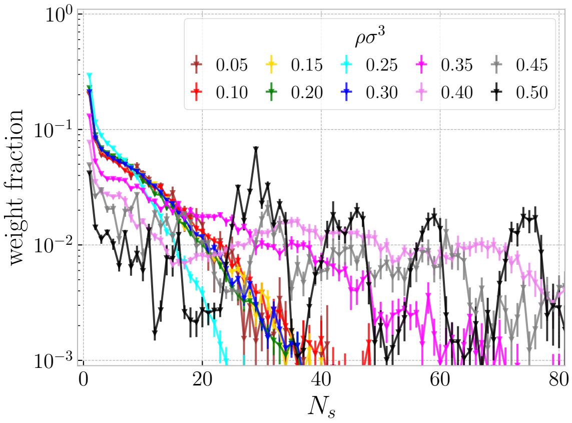

(d) charged rings, (+3) ions only

Figure S3: Weight fraction distributions of aggregation numbers, N_s, of rings in the emerging cylindrical stacks for different densities for counterion types.

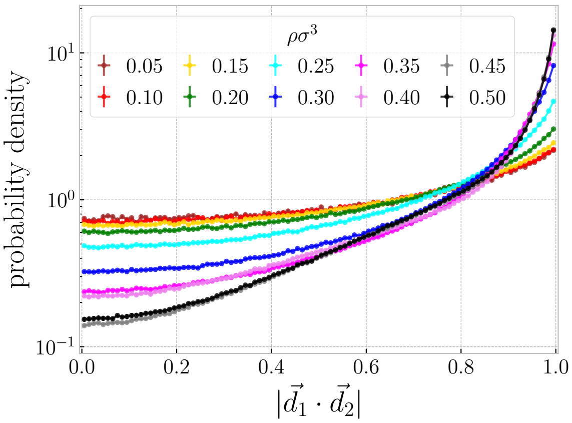

(a) neutral rings, no counterions

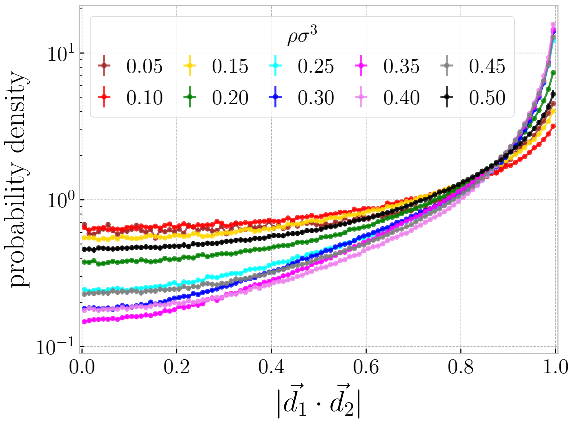

(b) charged rings, (+1) ions only

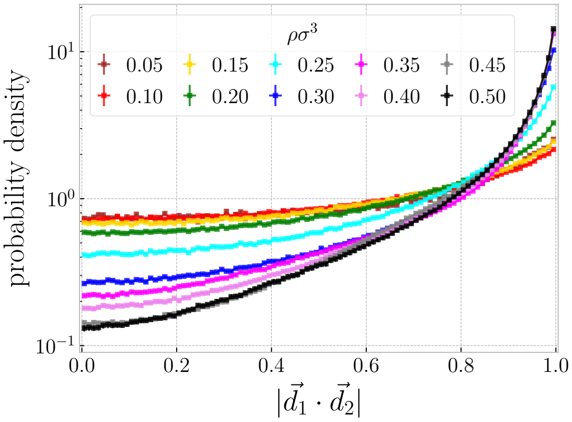

(c) charged rings, (+1)/(+3) ions

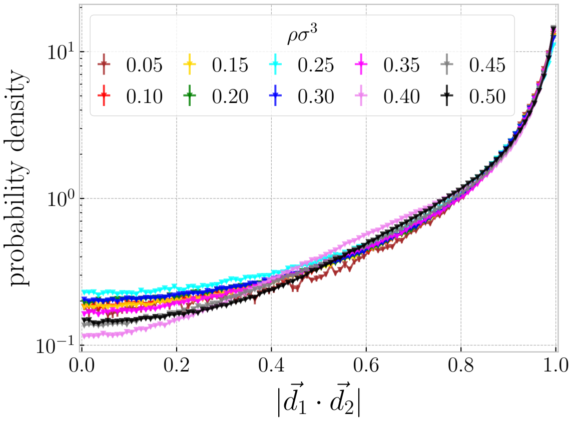

(d) charged rings, (+3) ions only

Figure S4: Probability distributions of mutual orientations of directors, $\vec{d}$, or pairs of rings separated by at most $0.5R_{\mathrm{g},0}$, plotted for different densities and counterion types.

S2 Single ring properties

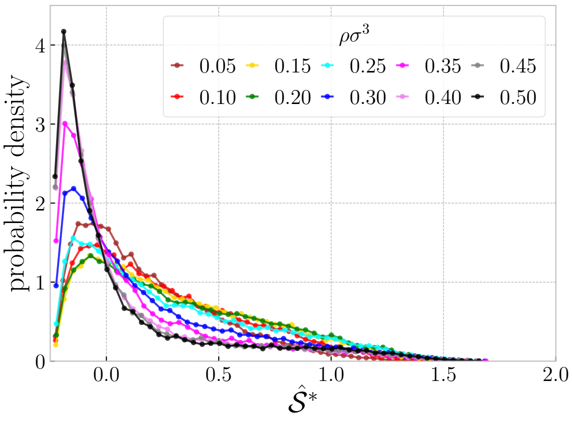

(a) neutral rings, no counterions

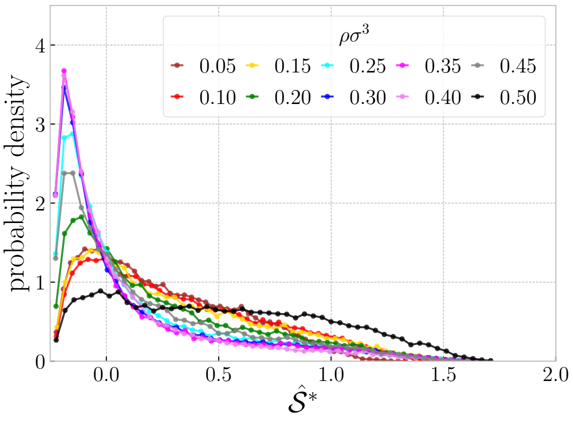

(b) charged rings, (+1) ions only

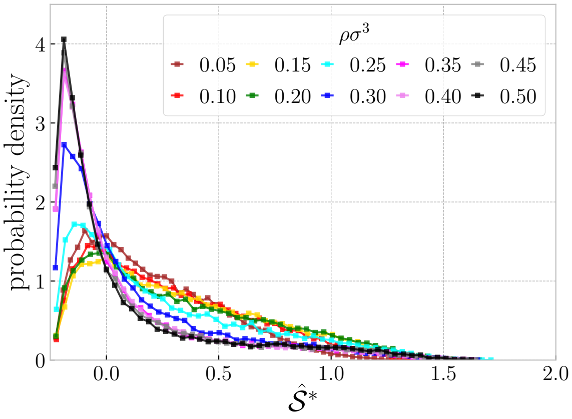

(c) charged rings, (+1)/(+3) ions

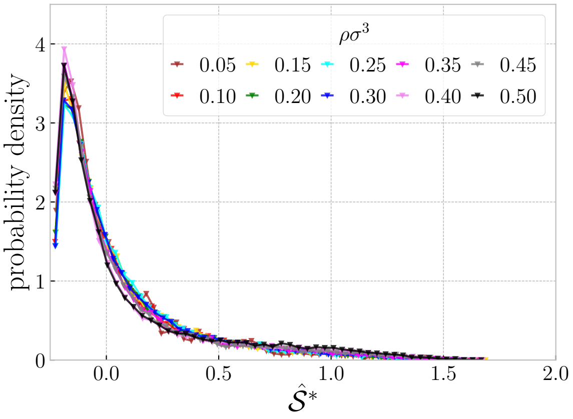

(d) charged rings, (+3) ions only

Figure S5: Probability distributions of prolateness of individual rings, plotted for different densities and counterion types.

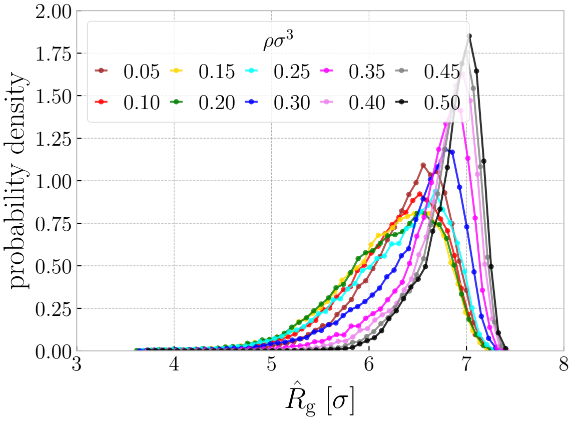

(a) neutral rings, no counterions

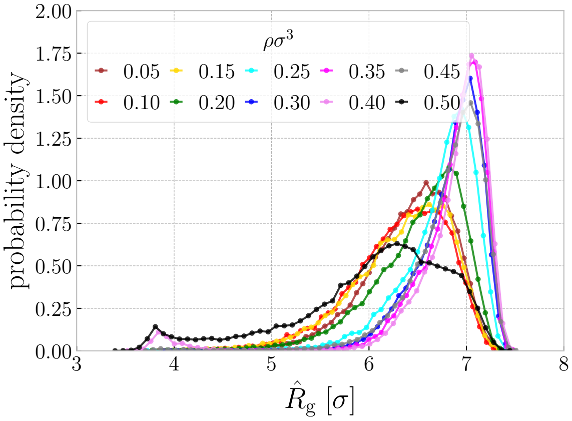

(b) charged rings, (+1) ions only

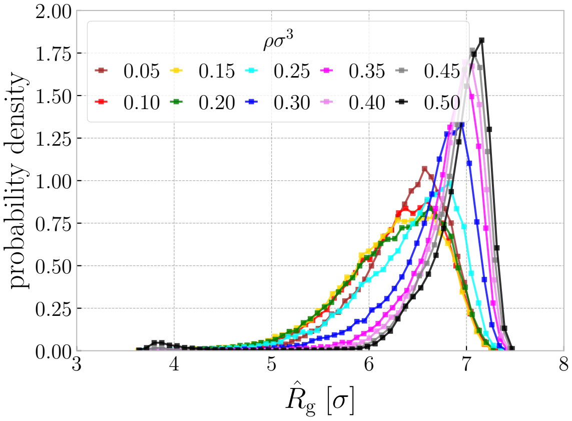

(c) charged rings, (+1)/(+3) ions

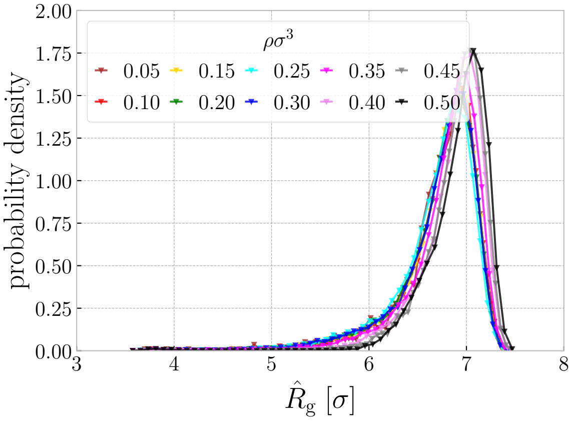

(d) charged rings, (+3) ions only

Figure S6: Probability distributions of radius of gyration of individual rings, plotted for different densities and counterion types.

S3 Role of counterions

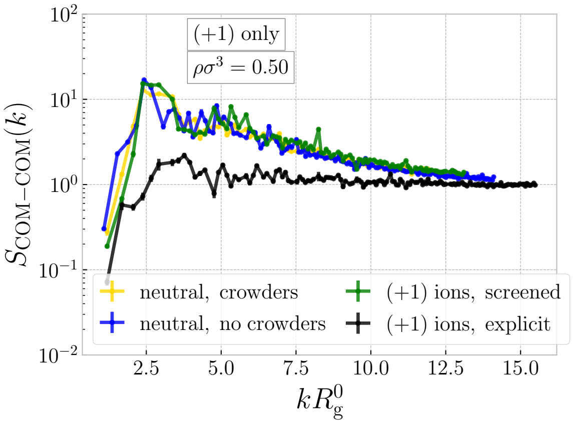

Figure S7: Static structure factor between centers of mass of the rings, for the system with monovalent ions only plotted for four different setups: neutral system with no counterions, neutral systems with neutral crowders, charged system with explicit electrostatics and charged system with screened electrostatics. The wavenumber is normalized by the radius of gyration of a single ring in infinite dilution.

S4 Dynamics

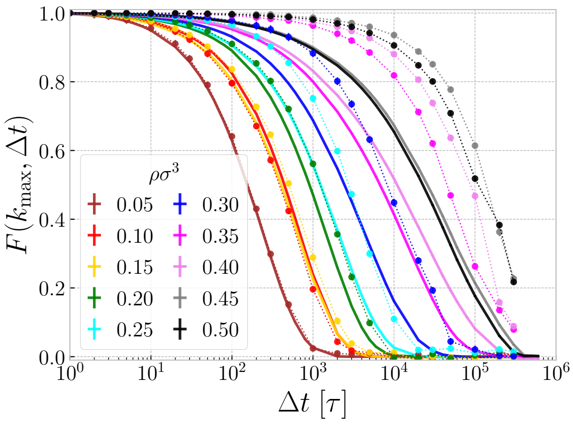

(a) neutral rings, no counterions

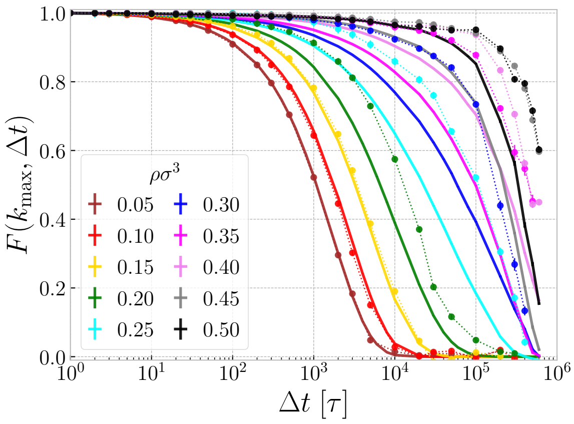

(b) charged rings, (+1) ions only

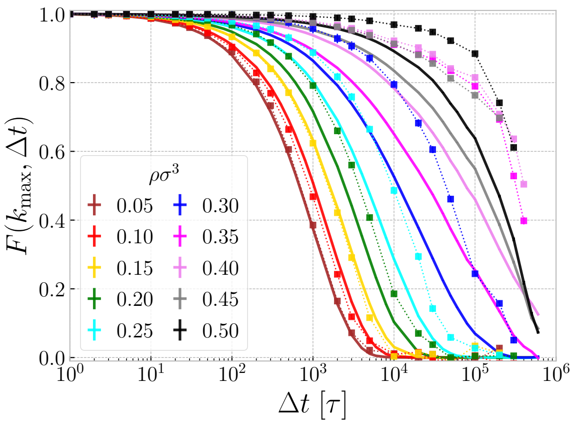

(c) charged rings, (+1)/(+3) ions

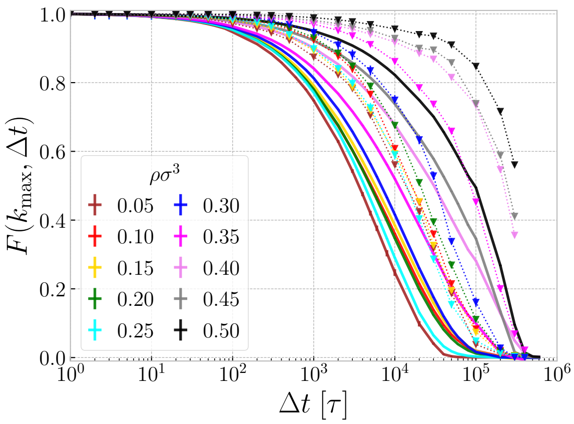

(d) charged rings, (+3) ions only

Figure S8: Intermediate scattering functions for centers of mass of the rings, for different densities and for the system with different ions. The incoherent (self) part is plotted with lines, while the coherent (collective) part with points and $k_{\max}$ is the wavenumber corresponding to the main clustering peak in Fig. S1.

S5 Threading analysis

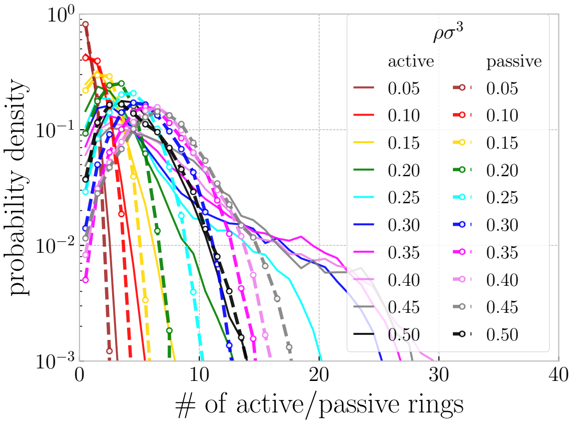

(a) neutral rings, no counterions

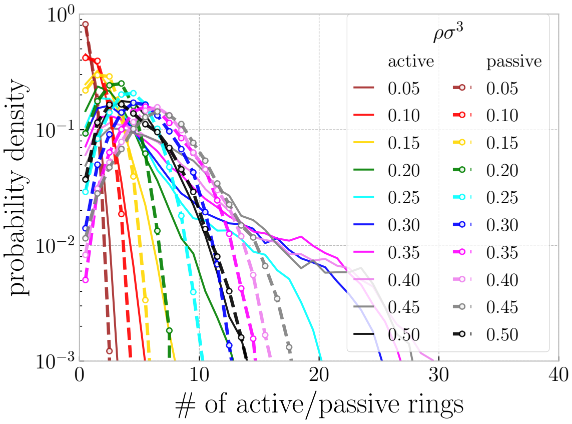

(b) charged rings, (+1) ions only

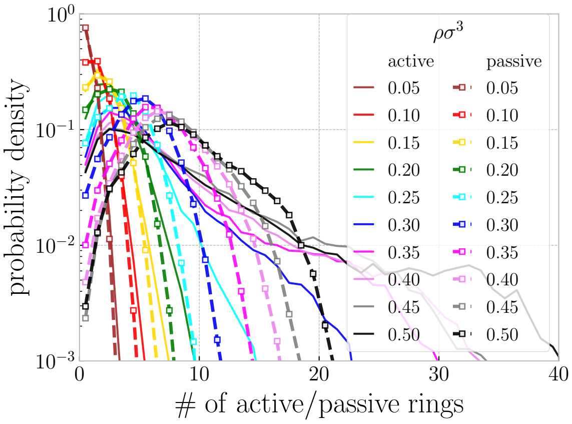

(c) charged rings, (+1)/(+3) ions

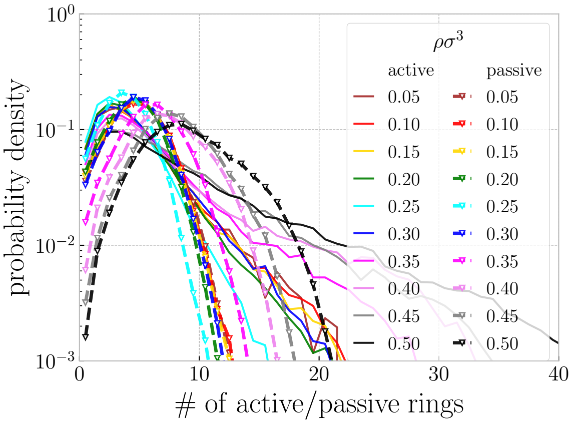

(d) charged rings, (+3) ions only

Figure S9: Probability distributions of number of passive partners (dashed line with points) and active partners (solid lines) per ring, plotted for different densities.

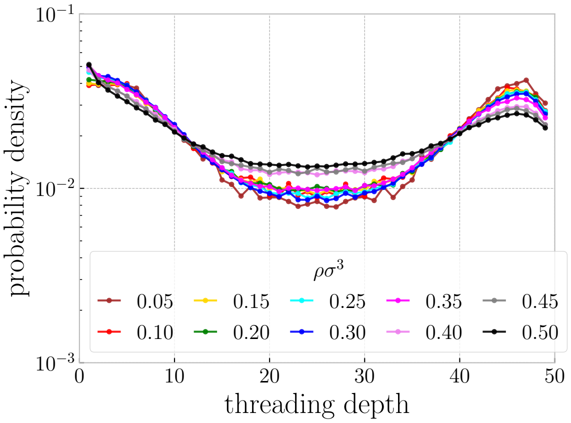

(a) neutral rings, no counterions

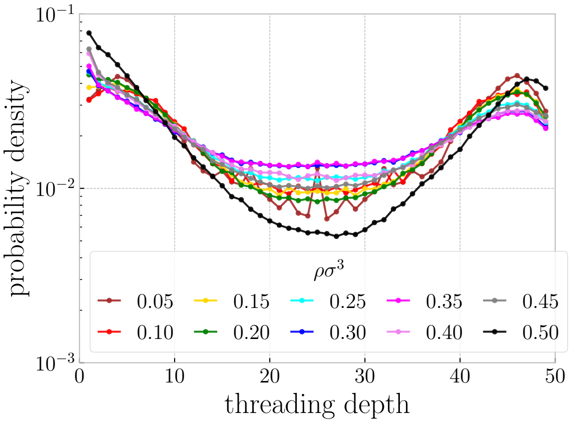

(b) charged rings, (+1) ions only

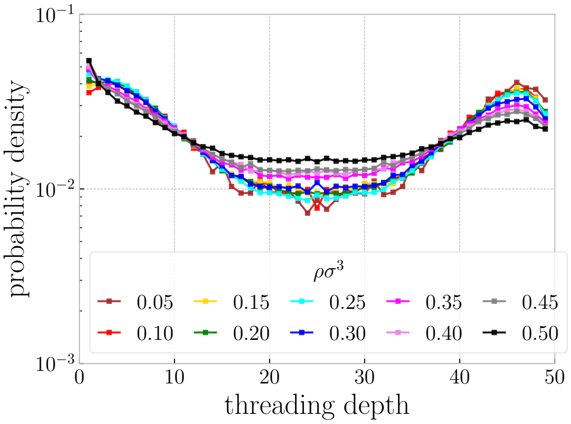

(c) charged rings, (+1)/(+3) ions

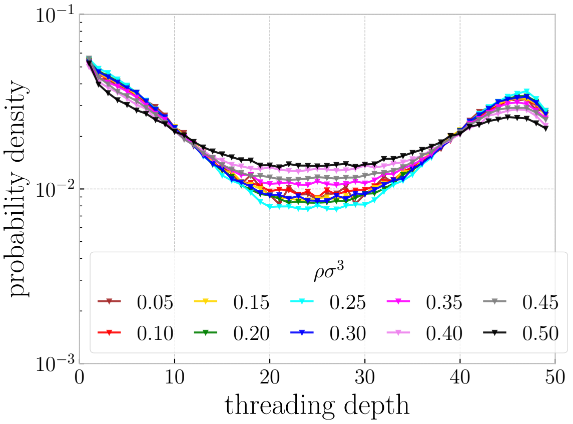

(d) charged rings, (+3) ions only

Figure S10: Probability distribution of threading depths for the active threaders.

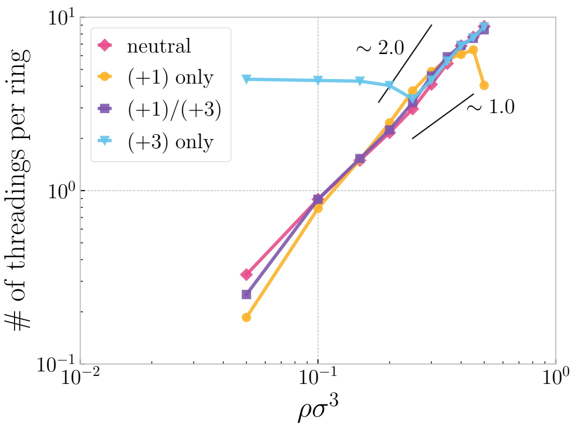

Figure S11: Number of threading partners per ring as a function of density for different ion valencies.
